# Recent Advances in the Discovery of SIRT1/2 Inhibitors via Computational Methods: A Perspective

**DOI:** 10.3390/ph17050601

**Published:** 2024-05-08

**Authors:** Naomi Scarano, Chiara Brullo, Francesca Musumeci, Enrico Millo, Santina Bruzzone, Silvia Schenone, Elena Cichero

**Affiliations:** 1 Department of Pharmacy, Section of Medicinal Chemistry, School of Medical and Pharmaceutical Sciences, University of Genoa, Viale Benedetto XV, 3, 16132 Genoa, Italy; naomi.scarano@edu.unige.it (N.S.); francesca.musumeci@unige.it (F.M.); silvia.schenone@unige.it (S.S.); 2 Department of Experimental Medicine, Section of Biochemistry, University of Genoa, Viale Benedetto XV 1, 16132 Genoa, Italy; enrico.millo@unige.it (E.M.); santina.bruzzone@unige.it (S.B.); 3 IRCCS Ospedale Policlinico San Martino, 16132 Genoa, Italy

**Keywords:** sirtuin, SIRT1, SIRT2, inhibitor, virtual screening, structure–activity relationship, computational methods

## Abstract

Sirtuins (SIRTs) are classified as class III histone deacetylases (HDACs), a family of enzymes that catalyze the removal of acetyl groups from the ε-N-acetyl lysine residues of histone proteins, thus counteracting the activity performed by histone acetyltransferares (HATs). Based on their involvement in different biological pathways, ranging from transcription to metabolism and genome stability, SIRT dysregulation was investigated in many diseases, such as cancer, neurodegenerative disorders, diabetes, and cardiovascular and autoimmune diseases. The elucidation of a consistent number of SIRT–ligand complexes helped to steer the identification of novel and more selective modulators. Due to the high diversity and quantity of the structural data thus far available, we reviewed some of the different ligands and structure-based methods that have recently been used to identify new promising SIRT1/2 modulators. The present review is structured into two sections: the first includes a comprehensive perspective of the successful computational approaches related to the discovery of SIRT1/2 inhibitors (SIRTIs); the second section deals with the most interesting SIRTIs that have recently appeared in the literature (from 2017). The data reported here are collected from different databases (SciFinder, Web of Science, Scopus, Google Scholar, and PubMed) using “SIRT”, “sirtuin”, and “sirtuin inhibitors” as keywords.

## 1. Introduction

Sirtuins (SIRTs) are classified as class III histone deacetylases (HDACs), a family of enzymes that catalyze the removal of acetyl groups from the ε-N-acetyl lysine residues of histone proteins, thus counteracting the activity performed by histone acetyltransferases (HATs) [1].

The dysregulation involving HDAC and HAT activities turns into different disorders [2]. Based on different subcellular localization and substrate specificity, SIRTs are classified in seven isoforms (SIRTs1-7). In addition, SIRT1, SIRT6, and SIRT7 are predominant as nuclear proteins, while SIRT2 and SIRT3-5 are mostly cytoplasmatic or placed in mitochondria, respectively [2,3].

Structurally, SIRT1-7 share a central catalytic domain of about 270 amino acids, and they are delimited by a Rossman fold and a smaller cavity with NAD^+^- and a zinc-binding site. However, the seven sirtuins differ in the N-terminal and C-terminal domains.

Extensive research has already been conducted on SIRT1 modulators, but also on SIRT2 [2,3], SIRT3 [4], and SIRT6 [5,6,7].

SIRT1 was the first human sirtuin to be identified. As it is involved in genome stability, stress response, and apoptosis events, its role in neurodegenerative disorders such as Parkinson’s disease (PD), Alzheimer’s disease (AD), and Huntington’s disease (HD) has been described in the literature [8]. In cancer, SIRT1 has a controversial role as it has been reported with oncopromoter and oncosuppressor functions [9].

SIRT2 is mainly expressed in the central nervous system (CNS) and, as a result, it is overexpressed in neurological pathologies, where it seems to promote neurodegenerative events [10]. Accordingly, SIRT2 inhibition is described as protecting neurons from toxicity due to increased α-synuclein levels, which is one hallmark in PD [11]. 

In AD, SIRT2 inhibition has been shown to reduce beta-amyloid converting enzyme 1 (BACE1) expression and improve cognitive impairment in AD mouse models [12]. In addition, SIRT2 expression is down- or up-regulated in different malignancies, thus making SIRT2 modulators interesting compounds in the search for anticancer agents.

This has aroused the interest of researchers around the world in developing sirtuin small molecule inhibitors (SIRTIs), or modulators that are not only used to treat different cancer types, but are also exploited in neurodegeneration and correlated pathologies.

**Figure 1:**
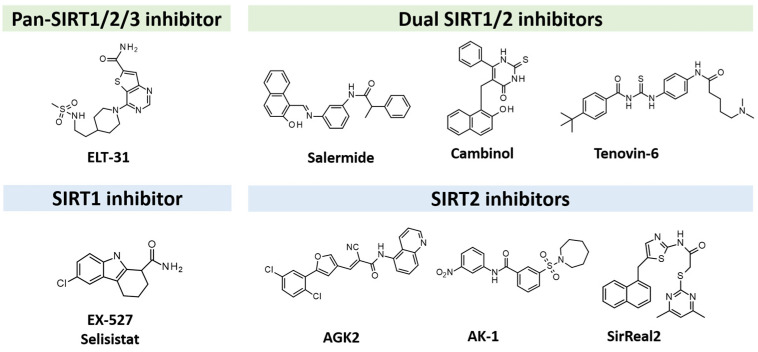
Chemical structure of the most-studied SIRTIs. Dual/pan SIRTIs are labeled in green while the selective ones are reported in cyan.

Up to now, several classes of SIRT1/2 modulators have been described. Among the inhibitors, **salermide** [13], **sirtinol** [14], **cambinol** [15], **suramin** [16], and **tenovin-6** [17] are dual SIRT1 and SIRT2 inhibitors, and they have been studied as antineoplastic, anti-proliferative, or as antiviral agents (Figure 1).

**AGK2** [18], **AK-1** [19], and **SirReal2** [20] are potent SIRT2Is that are able to prevent dopaminergic cell death, hippocampal neurodegeneration, or induce cell cycle arrest in human colon carcinoma cells. **ELT-31** is the most potent nanomolar pan-SIRT1/2/3 inhibitor, and it has been discovered by screening collections of chemo-typically diverse DNA-encoded small molecules [21], whereas **EX-527** (**selisistat**), a SIRT1I with IC_50_ in the nanomolar range, has been evaluated in clinical trials for the treatment of HD [22] and is now studied in phase II trials for endometriosis and other related diseases (**NCT04184323**).

Computer-aided drug design approaches, such as ligand-based and structure-based methods, represent widely exploited tools that are used to accelerate the drug discovery process [23,24]; in this context, the elucidation of a consistent number of SIRT–ligand complexes has been steering the identification of novel and more selective ligands. The individuation of selective isoform modulators represents a challenging task, especially for the high conservation observed in catalytic sites [25].

**Figure 2:**
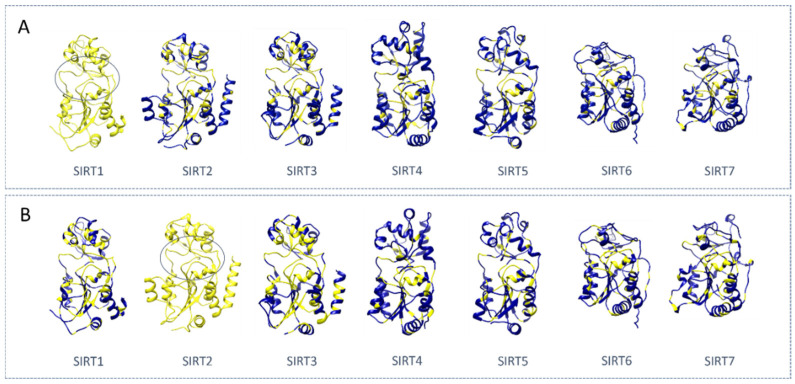
Conservation of the residues among the seven SIRTs with respect to SIRT1 (**A**) and SIRT2 (**B**). The non-conserved residues are colored in blue and the conserved residues are colored in yellow. High-sequence conservation was observed in the catalytic site (circled) of SIRTs1-3. Structure IDs: SIRT1:4IG9 [27], SIRT2: 3ZGO [28], SIRT3: 3GLS [29], SIRT4: AF-Q9Y6E7-F1 via AlphaFold [30,31], SIRT5: 4HDA [32], SIRT6: 5X16 [33], and SIRT7: AF-Q9NRC8-F1 via AlphaFold [30,31]. Note that, for SIRT4 and SIRT7, the predicted fold was used for the shown comparison in the absence of an experimental structure.

Based on sequence and structure alignment studies [25], SIRTs1-3 show a high sequence similarity and analog structural organization [26], while significant differences are observed if compared against SIRTs4-7 (Figure 2).

Due to the high diversity and quantity of the structural data thus far available, we reviewed the different ligand- and structure-based methods that have been recently used to identify new promising SIRT1/2 modulators. In particular, the present review focuses on two sections, including the information of SIRT1/2 to guide the search for novel selective ligands, and this is accompanied by computational studies leading to the discovery of SIRT1/2 modulators. The last section of this review deals with the most interesting SIRT1/2 inhibitor chemo-types that have recently appeared in the literature (from 2017), which were discovered through classical medicinal chemistry methods such as structure–activity relationship (SAR) exploration.

## 2. Structural Information of SIRT1-2

### 2.1. X-ray Crystallographic Structures of SIRT1

To date, eight crystal structures of *h*SIRT1 are available (see Table 1). The first structure was released in 2013, including an EX-527-based inhibitor, whereas the last was reported in 2015. The resolution values range from 1.85 Å (4KXQ) [27] to 3.2 Å (5BTR) [34]. Four structures contain the cofactor or a cofactor analog such as adenosine diphosphate ribose (ADPR), nicotinamide adenine dinucleotide (NAD^+^), and carbanicotinamide adenine dinucleotide (carba-NAD^+^), and five structures contain a modulator (or more), as shown in Table 1.

Four of the structures included activators bound to the N-terminal domain (or to part of it). Conversely, SIRT1Is were found to be bound at the catalytic site, specifically at the interface between the Zinc binding domain and the Rossmann fold. Two SIRT1-inhibitor complexes were reported, with one of them being a selective SIRT1I and the other a pan-SIRT1-3I [38]. Three of the structures exhibited a portion of the SIRT1 C-terminal regulatory segment (CTR), which was shown to stabilize the catalytic domain, thus enhancing its activity [27,37]. 

**Figure 3:**
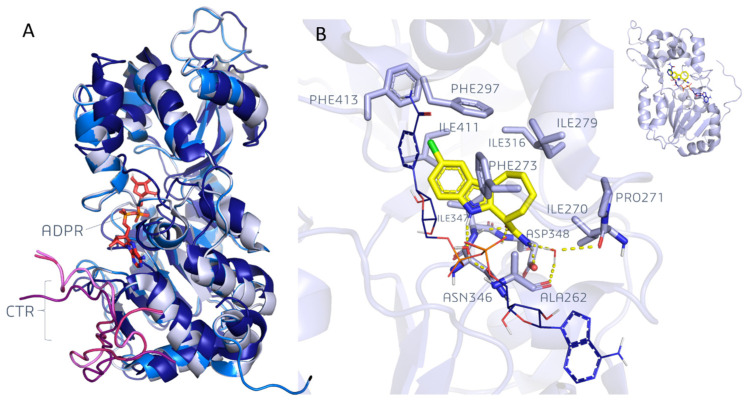
(**A**) Superimposition of the three apo-SIRT1s available on the Protein Data Bank (4IG9 [27]: dark blue, 4KXQ [27]: medium blue, and 4IF6 [35]: light blue). The ADPR in 4KXQ [27] is represented as sticks (dark orange), and the CTR as a cartoon (4IG9 CTR: dark violet, 4KXQ CTR: medium violet, and 4IF6 CTR: light violet). (**B**) The experimental binding mode of an EX-527 analog to the SIRT1 catalytic domain (PDB ID: 4I5I) [36]. The ligand is represented as sticks (yellow), and the cofactor (NAD^+^) is reported as lines (blue).

With respect to the apo-structures of SIRT1, a comparison of the closed-state PDB codes 4KXQ [27] and 4IF6 [35] revealed highly similar protein conformation (Figure 3A). The RMSD values at the corresponding main protein and CTR alpha carbon (CA) atoms were 0.09 Å and 0.34 Å, respectively, as estimated via MOE software MOE2019.01 [39].

Conversely, the superimposition of the open-state 4IG9 with the previous ones, 4IF6 [35] and 4KXQ [27], in the protein close state highlighted RMSD values at the main protein and CTR CA, spanning from 0.15 Å to 0.16 Å and from 3.08 Å to 3.09 Å, respectively.

In 2013, the role played by a synthetic SIRT1I (an EX-527 analog) was analyzed in the PDB code 4I5I [36] in the presence of the NAD^+^ cofactor. These experimental data highlighted the ligand–protein interactions within a mainly hydrophobic binding pocket, which was formed by PHE 413, PHE297, ILE279, ILE316, ILE441, ILE 347, PRO271, ALA262, PHE 273, and ILE 270 residues (Figure 3B). 

The amide moiety of the ligand established three direct H-bonds with ASP438 residues (i.e., sidechain and backbone amides), ILE347 (backbone) residues, and two water-mediated H-bonds with the backbones of ILE270 and ALA262. Another water molecule mediates the H-bond between the indole NH of the ligand and ASN346 sidechain. The NAD^+^ nicotinamide ring contributes to ligand stabilization through the non-polar contact with the Cl-phenyl moiety of the inhibitor [36].

**Figure 4:**
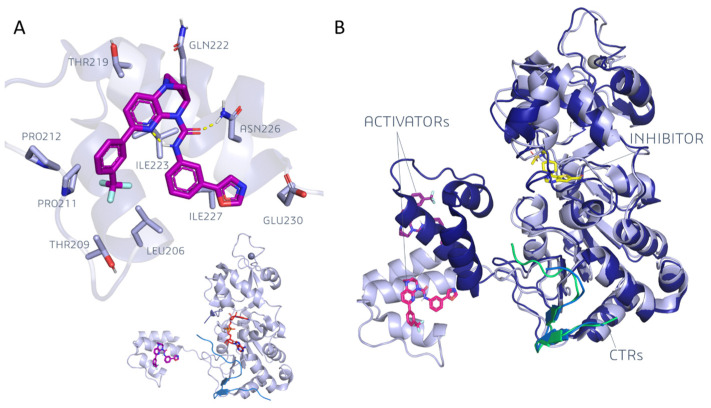
(**A**) The binding mode of a dihydropyridopyrazine derivative, a SIRT1 activator (PDB ID: 4ZZJ) [37]. The activator is bound at SIRT1 N-terminal domain. The catalytic domain was solved in a complex with a cofactor (NAD^+^, red sticks) and a substrate of the protein. The CTR fragment (light blue) is also reported. (**B**) The superimposition of the SIRT1 structure in complex with a diazepine derivative activator alone (magenta, light blue ribbon, and PBD ID: 4ZZH [37]) and the SIRT1 structure in complex with a dihydropyrido-pyrazine activator (purple) and a thienopyrimidine inhibitor (yellow, dark blue ribbon, and PBD ID: 4ZZI [37]). The CTR fragment is represented in green and in light blue for 4ZZH and 4ZZI, respectively.

In 2015, a SIRT1 activator was described in the 4ZZJ [37] PDB code in the presence of carba-NAD^+^, as bound to the surface of the N-terminal domain (Figure 4A). 

The carbonyl of the ligand establishes an H-bond with an ASN226 sidechain. Hydrophobic contacts are highlighted between the ligand and LEU206, THR209 (methyl), PRO211, PRO212, LEU215, and THR219 (methyl) residues, together with ILE223. Additionally, an intramolecular H-bond between the pyridine ring and the amide NH can be hypothesized [37]. In the absence of carba-NAD^+^, the 4ZZI [37] and 4ZZH [37] experimental data indicate the role played by the activator and inhibitor, and of the activator alone, respectively. 

As shown in Figure 4B, the N-terminal domain was placed in the proximity of the catalytic domain when in the presence of the inhibitor (4ZZI). Conversely, in the absence of the ligand (or substrate) the distance between the two domains was considerably increased. The relative position of the N-terminal domain with respect to the catalytic core has been the object of intensive studies as it is relevant to elucidate the activation mechanism of the target. Hydrogen–deuterium exchange mass spectrometry experiments have proved the coupling of the two binding sites, and the mutation of GLU230 and ARG446 residues have highlighted the key role of such residues in allosteric activation [37]. Nevertheless, such residues are far away from each other in the proposed structure, and the exact mechanism of activation has remained elusive. More recently, with the aid of computational tools, the SIRT1 activation mechanism was also investigated [40,41,42,43,44]. 

According to the most recent data [44], several different mechanisms of activation have been proposed depending on the interacting mode between the activators and substrates. Although, to our knowledge, no studies have reported the influence of inhibitor binding on the positioning of the N-terminal domain, it is possible to hypothesize that its presence would somehow condition the relative positioning of the two domains.

### 2.2. X-ray Crystallographic Structures of SIRT2

With respect to *h*SIRT2, thirty-seven crystal structures are available. The first structure was solved in 2001, the last in 2021. The resolution varies from 1.42 (4RMH) [45] to 3Å (5FYQ) [46], and it is available in sixteen structures containing a small-molecule inhibitor. 

Table 2 summarizes the available structural information for *h*SIRT2 as an apo-conformation. 

In several PDB IDs, only a SIRT2 substrate is associated to the catalytic domain of the apo-enzyme. In particular, myristoylated or acetylated peptide structures are reported.

**Figure 5:**
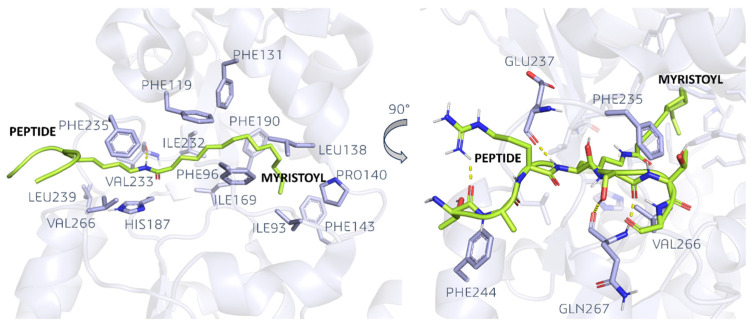
SIRT2 in complex with the H3K9myr substrate (PDB code = 4Y6L) [51]. The substrate is composed by a myristoyl portion, and it is linked via amide bond to a peptide group.

As an example, in PDB ID 4Y6L [51], the SIRT2 structure contains the myristoylated form of the H3K9 peptide (Figure 5). 

Myristoyl is inserted in a hydrophobic cavity located between the Rossman fold and the Zinc binding domain, which is formed by the VAL226, LEU239, HIS187, PHE235, PHE119, ILE232, ILE169, PHE96, PHE131, PHE190, LEU138, PRO140, PHE143, and ILE93 residues. The amide bond derived from the lysine myristoylation establishes an H-bond with the VAL233 backbone. The protein fragment to which myristoyl is tied to interacts with the entrance of the binding site by means of H-bonds with GLU237 (backbone) and GLN267 (backbone). Moreover, PHE235, PHE244, and VAL266 further stabilize the peptide. 

**Figure 6:**
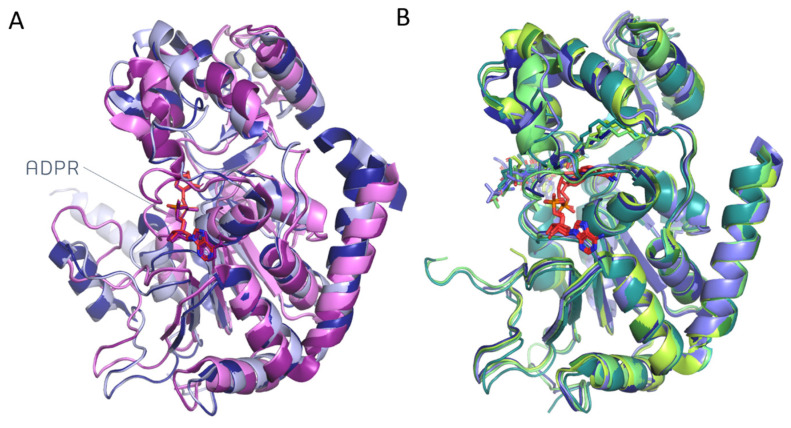
(**A**) Superimposition of the SIRT2 apo-structures lacking substrates/reaction products (1J8F [47]: dark blue, 3ZGO [28]: light blue, 3ZGV [28]: dark violet, ADPR in red sticks, and 5D7O [26]: light violet). ADPR is represented as sticks. (**B**) The superimposition of the substrate-bound structures of SIRT2 available on the Protein Data Bank (4Y6L [51]: forest, 4Y6O [51]: yellow-green, 5FYQ [46]: light-blue, 5G4C [49]: blue, 6L66 [50]: cold light green, and 6L65 [52]: teal). The cofactor, where present, is represented as red sticks.

A comparison of the available SIRT2 apo-structures in the absence and in the presence of ligands is reported in Figure 6A and Figure 6B, respectively.

Among the SIRT2 apo-conformations, i.e., 1J8F [47], 3ZGO [28], 3ZGV [28], and 5D7O [26], the first two types of experimental data for 1J8F and 3ZGO lacked cofactors and featured RMSD values at the corresponding CA of 0.61 Å. The 3ZGV and 5D7O included ADPR and proved to be highly similar after superimposition as the RMSD value at the corresponding CA atoms was 0.44 Å. Indeed, the ADPR positioning was fully maintained within the protein cavity as the two conformations were perfectly superposed.

In the absence of cofactors, the substrate-bound structures 4Y6L [51], 4Y6O [51], 5FYQ [46], and 6L65 [52] showed an RMSD spanning from 0.43 to 1.69 Å. While 4Y6L, 4Y6O, and 5FYQ were found to be highly similar (RMSD = 0.43–0.87 Å), the 6L65 conformation differs from the other ones based on the corresponding RMSD values (RMSD = 1.58–1.69 Å). The additional presence of carba-NAD^+^ or NAD^+^, as featured by 5G4C [49] and 6L66 [50], led to highly comparable SIRT2 conformations with a corresponding RMSD value of 0.70 Å.

Through probing SIRT2-inhibitor complexes (holo-forms), the presence of a peptide-based inhibitor was initially considered (PDB code = 4L3O) [55]. A perspective of this piece of information is summarized in Table 3.

In addition to the macrocyclic inhibitor that is in complex with SIRT2 and showed in 4L3O [55], several reaction mechanism-based inhibitors (e.g., thio-myristoyl peptides), as shown in the 4R8M [59], 4X3P [48], 6NR0 [62], 7BOS [64], and 7BOT [64] PDB codes, have been taken into account. These experimental data have been exploited to investigate the catalytic mechanism as they were shown to slow down the reaction, thud making it easier to solve the reaction intermediates. NAD^+^ or NAD^+^ analogs were found to be present in the majority of the cases. 

Regarding the non-reaction mechanism related to SIRT2Is, most of the reported X-crystallographic data contain an SIRT2 isoform-selective ligand. Indeed, most of the chemical diversity thus far explored is limited to the SirReal2 series, as previously reported by us [66] and confirmed by the abundance of X-ray SIRT2 structures complexes containing **SirReal2** or its derivatives (see Table 3).

**Figure 7:**
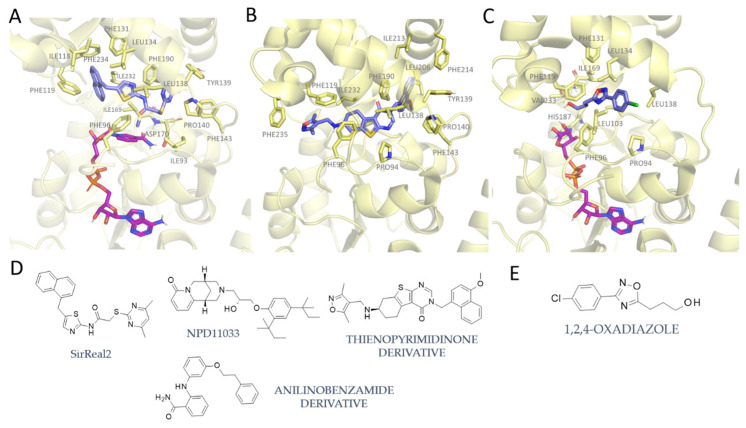
The experimental data of (**A**) **SirReal2** in 4RMG [45] and of (**B**) a thienopyrimidinone compound in 5MAT [56]. (**C**) The binding mode of the oxadiazole inhibitor in complex with SIRT2 (PDB code = 5MAR) [65]. Slate blue: ligands, pale yellow: protein, and purple: cofactor. (**D**) The chemical structure of compounds inducing the so-called “selectivity pocket”, such as SirReal2, **NPD11033**, an anilinobenzamide derivative, and the 5MAT thienopyrimidinone co-crystallized ligand. (**E**) The chemical structure of the 5MAR co-crystallized ligand.

As shown in Figure 7A, **SirReal2** occupies the binding pocket at the interface between the Rossman fold and the Zinc binding domain (PDB code = 4RMG) [45]. 

The ligand is mainly hydrophobic and forms non-polar interactions within the binding pocket (ILE118, PHE234, LEU138, TYR139, PRO140, PHE143, ILE93, ILE232, PHE96, ILE169, PHE190, and PHE119). Of particular relevance is the π–π stacking interaction between the pyrimidine ring of **SirReal2** and PHE190, which exhibits an advantageous staggered position. PHE119 instead interacts orthogonally with the naphthalene moiety of the ligand. An intramolecular H-bond involving the amide function and the pyrimidine ring confers rigidity to the ligand (PDB ID: 4RMG) [45].

Selective SIRT2Is include thienopyrimidinone-derived inhibitors. The binding pose of a thienopyrimidinone to SIRT2 is reported in Figure 7B (PDB ID: 5MAT [56]). The ligand establishes several hydrophobic contacts with the non-polar residues of the binding site (PHE235, PHE96, ILE232, PRO94, PRO140, PHE143, LEU206, ILE213, PHE214, ILE169, LEU138, PHE190, and TYR139). The latter interacts orthogonally with the naphthalene moiety of the ligand that, on the other side, forms a π-stacking interaction with PHE190. For this particular inhibitor, no H-bonds were highlighted.

This piece of information is of capital importance in the rationalization of isoform selectivity, and it has been previously investigated on the basis of key residues at the binding site [25]. According to this study, the SirReal2 ligand achieves selectivity with respect to other sirtuins through induced-fit effects. In particular, the ligand is able to cause conformational changes in the active site, thus expanding the volume of the binding pocket to create an additional sub-pocket—the so-called “selectivity pocket”. This pocket exhibits unique features with respect to SIRT1 and SIRT3 [45], thereby offering a possible explanation of the selective behavior of the SirReal2 series.

The formation of the selectivity pocket was also observed for other classes of selective compounds, namely **NPD11033** [58], thienopyrimidinone-derived inhibitors [56], and anilinobenzamide derivatives [20] (see chemical structure in Figure 7D).

Indeed, the 5MAT [56] thienopyrimidinone-based ligand has proved to occupy the same enzyme cavity that was previously described for **SirReal2** (Figure 7A) as it is engaged in π–π stacking with PHE131 and PHE143 and is H-bonded to TYR139 (Figure 7B).

Conversely, 1,2,4-oxadiazoles (Figure 7C) exhibit a distinct inhibition mechanism [65] that does not comprise the block of SIRT2 in the locked-open conformation.

As shown in Figure 7C, the oxadiazole can bind the active conformation of the target, in which the zinc binding domain is rotated and “closed” with respect to the **SirReal2** complex. These compounds are, in fact, uncompetitive inhibitors with respect to substrates and cofactors, and they occupy a sub-cavity in the proximity of LEU134 and LEU138 [65]. 

## 3. Computational Methods toward the Discovery of SIRT1-2 Modulators

### 3.1. Design of SIRT1 Modulators via Computational Approaches

The first application of virtual screening (VS) in SIRT1 drug discovery was reported in **2008** by Huhtiniemi et al. [67], who used a previously reported structure-based (SB) pharmacophore model to screen compounds from the Maybridge and Leadquest databases [68]. As no crystal structures of the target were available at that time, the model was built on the basis of in silico-derived information. The subsequent in vitro tests on SIRT1 and SIRT2 led to the individuation of a novel oxadiazole-carbonylaminothiourea scaffold that is active in the micromolar range. The most potent compound (see Table 4, Entry 1) showed moderate selectivity for SIRT1 over SIRT2 (IC_50_ of 113 μM for SIRT2 VS 13 μM for SIRT1), while all the other compounds were non-selective. The molecular docking studies of the compound performed at the SIRT1 binding site indicated a key role played by the oxadiazole ring in being H-bonded to the enzyme ILE347 backbone. No docking studies have been reported involving the SIRT2 protein.

An innovative sequence-based approach was developed by Wang et al. [69] in **2011**. They built and trained a support vector machine (SVM) with a set of protein–ligand interactions, which were observed for hundreds of different targets. This general (target-unspecific) model was used to predict the protein–ligand interactions of SIRT1 (using the sequence as input) and a set of ligands by the SPECS drug-like library [70]. A number of one hundred and sixty-five candidates were obtained and tested in vitro, and five of them were active in the 5–50 μM range (see, as an example, Table 4, Entry 2).

**Table 4:** A perspective of the successful computational studies applied in the search of SIRT1 modulators. The activators (A) and inhibitors (I) are listed. Structure-based virtual screening (SBVS) studies and ligand-based (LB) ones, or LB/SBVS, are highlighted in light cyan and light yellow. The release date (R.D.) and the corresponding reference (Ref.) are reported.

Entry	R.D.	Ref.	Type of Computational Method	Selectivity over Other Isoforms	Screened Database (n. of Compounds)	Most Active Compound/Proposed Compound	Activator/ Inhibitor	Potency
1	2008	[67]	Structure-based–Pharmacophore-based	1,2	The Maybridge and Leadquest libraries	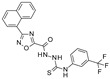 Oxadiazole-carbonylaminothiourea	I	13 μM (IC_50_, SIRT1)
2	2011	[69]	Sequence-based VS	Not tested	SPECS drug-like library (85,000)	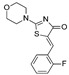 Various	I	5.72 μM (IC_50_)
3	2012	[71]	Target: HM	Not tested	In-house database (2500)	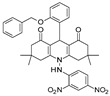 Acridinedione derivatives	I	0.25 μM (IC_50_)
4	2014	[72]	Target model for inhibitors: crystal structure; Target model for activators: HM of the allosteric site	Not tested	Asinex (>600,000)	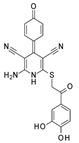	I	16.35 μM (IC_50_)
5	2016	[73]	SBVS	Not tested	Drug bank library from ZINC (1716)	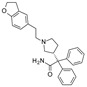 Diphenyl and oxycoumarin derivatives	I	77.7% inhibition at 5 μM
6	2020	[74]	Iterative in vitro and in silico screenings	2,3,5	Small library of previously identified putative SIRT-1 inhibitors	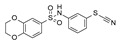 Thiocyanates	I	5.2 μM (IC_50_)
7	2021	[75]	SBVS	Not tested	In-house library (54)	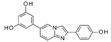	A	79% at 100 μM
8	2022	[76]	SBVS	Not tested	In-house library of 1,8-dioxo-octahydroxanthene derivatives (18)	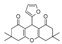	I	87.6% at 10 μM
9	2022	[77]	SBVS	2,3	In-house library (1,000,000)	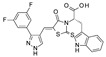	I	0.66 μM
10	2023	[78]	SBVS	2,3	SPECS database	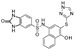 **hsa62**	I	1.3 μM (IC_50_)
11	2016	[79]	LB and SB pharmacophore models combined with docking-based VS	Not tested	ASINEX (5 000 000), in-house (971)	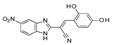 Benzimidazole derivative	I	4.34 μM (IC_50_)
12	2019	[80]	LB and SB pharmacophore models	Not tested	Indonesian Herbal Database (1377)	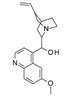 Mulberrin, **quinine**, quinidine, and gartanin	A	1.14 μM (EC_50_)
13	2019	[81]	Contest-based approach (combined methods)	Not tested	ENAMINE (2 459 912)	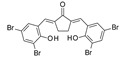 various	I	4.1 μM (IC_50_)
14	2022	[82]	LB and SB methods	Not tested	In-house library	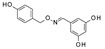	A	62.3% at 100 μM

A homology model of the SIRT1 catalytic domain was instead used by Alvala et al. [71] in 2012 to screen an in-house database of 2500 compounds. The optimization of the newly discovered acridinedione led to a hit compound with activity in the sub micromolar range and it also showed anticancer potential (see Table 4, Entry 3). In particular, antitumor studies of the identified inhibitor showed a dose-dependent increase in the acetylation of p53K382 and a decrease in the SIRT1 in MDA-MB231 breast cancer cell lines. Through a 3D-QSAR analysis, the key role played by hydrophobic and non-polar tethers to the prototype was identified. No experimental selectivity studies have been reported. 

In 2013, the first crystal structure of SIRT1 was solved, thus making it possible to perform SBVS on the crystal structure itself (PDB ID = 3ZGO) [28]. 

In 2014, the Pulla Alvala group screened the Asinex database [83] against the crystal structure of SIRT-1 in complex with an analog of SIRT1I **EX-527** and NAD^+^ (PDB code 4I5I) [72]. Docking studies have been performed on the amino acids from 180 to 220 as key residues for binding based on their relevance in modulating SIRT1 through allosteric interaction, as suggested by mutation studies. This kind of approach has led to the individuation of two promising SIRT1Is with IC_50_ values around the 16–32 μM range (see Table 4, Entry 4), as well as of a novel activator [72]. Comparative docking evaluation has revealed the H-bonds between the inhibitor and GLN345, ILE347, and ASP348, while contacts involving SER174, ASP175, ARG167, GLU190, and the activator have also been detected. 

In 2016, Padmanabhan et al. [73] selected the hSIRT1/ADPR co-crystal (PDB ID: 4KXQ) [27] to perform a SBVS of a Drug Bank [84] set of compounds. The resulting diphenyl-oxycoumarin derivatives were further investigated by means of MD simulations, which were then experimentally evaluated. Four compounds exhibited inhibitory activity in vitro (see, as an example, Table 4, Entry 5). 

In 2020, an iterative in silico–in vitro procedure was adopted by Wössner and colleagues [74] with the aim of individuating novel SIRT1Is. The crystal structure of SIRT1 in complex with **EX-527** and NAD^+^ (PDB ID 4I5I) [36] was utilized to screen a small library of compounds. The simulation output was evaluated in vitro, thereby validating the bioactivity of a thienopyrimidone-thiocyanate scaffold (see Table 4, Entry 6). 

To further optimize these compounds, one-hundred and thirteen thiocyanate-containing molecules from the Princeton BioMolecular Research Compound collection [85] were docked in the SIRT1 active site. In vitro tests of the most promising candidates showed an ameliorated activity of the optimized compounds (IC_50_ values in the low micromolar range), with most of them exhibiting the thiomethyl-amide main group. Moreover, high selectivity with respect to SIRT2-3 was also demonstrated.

In 2021, Flori et al. designed an in-house library of fifty-four putative SIRT1 activators [75], and they then screened these compounds against a SIRT1 X-ray structure, which hosted three molecules of resveratrol (PDB ID: 5BTR) [34]. The binding site was defined around all of the three possible crystallographic sites of resveratrol. Molecule selection was performed on the basis of the calculated binding free energy and predicted pharmacokinetics and cardiotoxicity, taking resveratrol itself as a reference compound. Eleven compounds, including different imidazopyridines, were tested in vitro, and five out of the eleven screened derivatives showed activity toward the target. The most promising compound (see Table 4, Entry 7), an imidazopyridine tethered to two terminal phenolic rings, was also tested ex vivo to assess its cardioprotective potential (see Table 4, Entry 7). 

More recently (2022), a small-scale VS of a series of 1,8-dioxo-octahydroxanthene derivatives was performed by Manikanttha et al. on the SIRT1 catalytic domain [76]. This class of derivatives was reported to be promising in cancer therapy by tests on several cell lines, and similar compounds were already reported as potentially active on sirtuins [86,87,88]. On this basis, a set of eighteen analogs were synthesized and evaluated in silico using 4I5I as a docking template. The results were visually inspected, and four compounds were chosen for in vitro tests on SIRT1. Among them, one (see Table 4, Entry 8) showed an encouraging inhibition (>87%) of SIRT1 at a 10 μM concentration. 

As previously mentioned, Purushotham et al. reported a VS of approximately 1 million compounds toward the **EX-527**-SIRT-1 co-crystal (PDB ID: 4ZZI) [37], thereby leading to different scaffold featuring SIRT1 inhibitory ability, such as the benzimidazole motif and further heterocyclic rings [77]. Drug-like filters were applied to select the best derivatives, and the top three compounds were selected for further study. Two promising scaffolds for SIRT1 inhibition (benzimidazole and pyrazole) were then conjugated with amino acidic fragments, thereby producing twenty-four novel candidates with desirable pharmacokinetics and low toxicity. These molecules were submitted for in vitro evaluation, and the best nine were tested in cellular tests. Four compounds were proposed as comparable to inhibitors of **EX-527** as they exhibited ameliorated selectivity with respect to the prototype. The most interesting derivative of this series is represented in Table 4, Entry 9. The authors also evaluated their selectivity, thus revealing the key role played by electrostatic interactions with the “SLxVxP(V/F)A” motif. This is located at the lower cleft of the substrate binding site.

In 2023, Cai and colleagues carried out VS calculations based on the experimental data of SIRT1 (PDB code = 4I5I) [36] in the presence of the **EX-527** inhibitor [78]. The authors used the SPECS database (http://www.specs.net), which contains structural information related to 100,000 chemicals, for the desired VS calculations using Glide software (Schrödinger suite 2020, www.schrodinger.com). Among the selected molecules, those exhibiting a SIRT1 inhibition rate above 50% at 10 μM in enzymatic assays were selected for further IC_50_ value determination.

A number of naphthyl-based derivatives proved to be effective as SIRT1Is, being compound **hsa62** (see Table 4, entry 10) the most promising (SIRT1 IC_50_ = 1.3 μM). Following a selectivity evaluation of SIRT2-3, the ligand promiscuous inhibitory ability was revealed (SIRT2 = IC_50_ 5.5 μM, SIRT3 IC_50_ = 43.6 μM) [78].

In other cases, the SBVS approach was combined with ligand-based (LB) techniques. In a 2016 study, Pulla et al. [79] developed an energy-based pharmacophore model on the basis of the SIRT1/**EX-527**/NAD^+^ co-crystal (PDB code: 4I5I) [36] in addition to a ligand-based pharmacophore model. This was built on a set of seventy-nine known active ligands. The pharmacophore features were used to pre-filter a large database, and the results were docked in the SIRT1 active site following a multi-step protocol. In vitro tests individuated a novel class of benzimidazole-containing compounds, of which the most potent exhibited an IC_50_ of 4.34 μM (see Table 4, Entry 11). This showed the attenuation of the proliferation of prostate cancer cells (LnCAP), as well as a reduction in the production of reactive oxygen species. This event also entailed a reduction in pro inflammatory cytokines such as IL6 and TNF-α. The anti-inflammatory potential of this derivative was investigated using an animal paw inflammation model induced by carrageenan, thus giving further support to the development of this series of SIRT1Is to treat both cancer and inflammation.

Finally, in 2023, structure-based studies conducted by Shen et al. allowed for the identification of **Tipranavir** as a SIRT1I with anti-hepatocarcinoma activity [89]. In particular, it was found that **Tipranavir** selectively inhibits HepG2 cell proliferation without causing toxicity to normal human hepatic cells, thus also causing a SIRT1 expression reduction. A mouse xenograft tumor model was exploited to ascertain the antitumor ability of **tripanavir**. The compound treated mice showed a 39.6% reduction in tumors when compared to the control mice. The stability of the SIRT1–**tripanavir** complex has been thoroughly evaluated via MD simulations. The results have indicated that PHE414, PHE273, and PHE297, and VAL412 and PHE414 are predominantly involved in hydrophobic contacts and H-bonds with the ligand, respectively.

With respect to the discovery of new SIRT1 activators [80], several efforts were performed by the Azminah group in 2019. The co-crystal of SIRT1 in complex with a small molecule activator (PDB code: 4ZZJ) [37] was used to generate a SB pharmacophore model. Moreover, an LB pharmacophore was modeled. Both the models were used to separately screen a set of natural compounds by the HerbalDB [90]. LB screening retrieved **quinine**, **quinidine**, and **gartarin** as the best hits, while the SB model individuated **mulberrin** as the putative active compound (see Table 4, Entry 12). In particular, the molecular docking studies showed the ILE223 and ILE227 at the allosteric region as the most important residues involved in the ligand binding. The selected compounds were confirmed to be active by means of in vitro tests.

A large-scale contest-based study was reported by Chiba [81], which allowed for testing and comparing different in silico strategies for the individuation of SIRT1Is. The study involved sixteen groups and led to the discovery of seven structurally distinct hits. Among them, the most promising, **Z56773446**, is shown in Table 4, Entry 13. The rate of success of that study was calculated: among 3192 were assayed and 0.13% of the compounds were endowed with SIRT1I inhibitory ability.

In 2022, the resveratrol phenolic portion was investigated via in silico studies, which led to the identification of SIRT1 activators exhibiting the methyleneoxyamine linker tethering of the resveratrol terminal aromatic rings and a further aromatic core [82]. The results revealed the most promising derivative as endowed with a modest SIRT1 activating ability (SIRT1 activity = 62.8%) (Table 4, Entry 14). Despite this, the antioxidant effects of this hit compound in human umbilical vein endothelial cells (HUVECs) injured with H_2_O_2_ at 100 µM was determined. This discovered SIRT1 activator significantly preserves cell viability and prevents an intracellular reactive oxygen species increase in HUVECs exposed to oxidative stimuli. This result was quite comparable with that of the reference activator resveratrol, thus suggesting a higher ability of the proposed hit compound to pass cell membranes and to activate the intracellular SIRT1.

### 3.2. Design of SIRT2 Inhibitors via Computational Approaches

Prior to the publication of the first inhibitor-bound SIRT2 crystal structure in 2015 (PDB ID 4RMG) [45], SBVS strategies were often coupled to MD simulation to generate in silico protein conformations, which is suitable for inhibitor accommodation. This approach was successfully followed by Tervo et al. [91] in 2004. The research group submitted to MD the available apo-form of SIRT-2 (PDB code 1J8F) [47], and they extracted an artificial enlarged conformation of the target. The binding pocket interaction fields and properties were calculated and used to screen the Maybridge database [92]. Docking of the most promising compounds and in vitro tests led to the individuation of five active phenolic-containing compounds. In particular, the docking mode of **sirtinol** as a reference and flexible inhibitor was taken into account and compared with those of the screened derivatives. A common feature of all the derived conformations was the ability of the aromatic ring moieties to interact via hydrophobic contacts with PRO115, PHE119, LEU138, ILE169, PHE190, and ILE232. On the other hand, hydrogen bonds with the carboxyl group of ASP95 were also detected. This piece of information allowed for highlighting the main hydrophobic and H-bond contacts that can be exploited for the development of optimized phenolic-based SIRT2Is. 

Among the studied compounds, two of the five molecules yielded in vitro IC_50_ values of 56.7 and 74.3 μM, with the most promising shown in Table 5, Entry 1.

In 2006, the same authors used a similar approach [93]: an MD-derived conformation. This was generated by the apo-form of SIRT2. The docking of known inhibitors allowed for the hypothesizing of key protein–ligand interactions for binding, which were used as queries to screen compounds from the Maybridge Screening Collection [92] and the Lead Quest databases. A set of eleven hits was tested in vitro, and four derivatives inhibited SIRT2 at micromolar concentrations. A novel indole-like structure (which has the code name **TRIPOS 360702** in the LeadQuest database) tethered to the main piperidine ring was identified as a promising scaffold for the development of SIRT2Is (see Table 5, Entry 2).

Accordingly, the same authors published further optimized derivatives [103] while also bearing in mind the structural requirements needed to achieve SIRT2 inhibition, as highlighted in their aforementioned SB studies [91,93], where a series of analogs were designed to explore the compound molecular size in affecting sirtuin inhibition. Structural substitutions involving the previous **TRIPOS 360702** terminal phenyl group and indole moieties have been considered, as well as variations at the main piperidine group. The results led to compounds that are equipotent to the prototype and endowed with higher SIRT2/SIRT1 selectivity values [103].

Successively, further efforts reported in the literature have allowed the discovery of a novel series of indole-based derivatives in association with an additional triazole moiety [104] that exhibit different selectivity profiles as SIRT1 and/or SIRT2Is. While four compounds have proven to be specific for SIRT1 inhibition, three were selective SIRT2Is and two were dual SIRT1 and SIRT2 inhibitors.

In 2010, Sivaraman [94] performed an SBVS on the apo-form of SIRT2 (PDB code 1J8F) [47] using an NCI Diversity Set as the screening library [105]. The selected compounds were experimentally tested, and a new nucleoside-like inhibitor of SIRT2 was unveiled. The hit compound selectivity over SIRT1 was evaluated featuring a moderate preference for SIRT2 over SIRT1 (with an inhibition of 71% and 20% at 50 μM, respectively). As shown in Table 5 (Entry 3), the **NSC671136** compound has a thiopyrimidinone-based structure, and it experiences stronger inhibitory activity toward SIRT2 (IC_50_ = 8.7 μM) over SIRT1. Based on the reported molecular docking studies, the ligand binds to the SIRT2 protein at the acetylated peptide binding pocket, thereby moving the phenyl rings in the proximity of the PHE235- and PHE244-detecting hydrophobic contacts. The main pyrimidinone core was engaged in further contacts with LEU239, while the dichlorobenzoate moiety displayed polar interactions with the non-conserved GLN267, SER271, and LYS275 residues.

In 2011, Schlicker et al. [95] performed parallel VS experiments on several SIRTs (SIRT2 (1J8F) [47], -3 (3GLS) [29], -5(2NYR) [106], and -6 (3K35) [107]) with the aim to identify isoform-specific novel inhibitors. An ADPR molecule was introduced in the SIRT2, 3, and 5 structures using the SIRT6/ADPR complex as a reference (PDB code: 3K35) [107]. The National Cancer Institute (NCI) [105] diversity set was used as a screening library. In vitro experiments of the top compounds confirmed the activity of twenty candidates that exhibited different chemo-types. Based on following assays, fourteen compounds proved to be selective for SIRT2. The chemical structure of **CSC8** is reported in Table 5, Entry 4. In the model of the SIRT2-**CSC8** complex, hydrophobic interactions were observed between the estradiol ring system and PHE96, LEU107, PHE119, and ILE169, which belong to a deep protein hydrophobic cavity that extends into the small Zn^2+^-domain. In addition, the hydroxyl group of the hit was H-bonded to ASN168 and GLN167. Following this approach, semi-specific and non-specific inhibitors were also retrieved.

A few years later, the crystal structure of SIRT2 in complex with ADPR (PDB code 3ZGV) [28] was used by Sacconnay et al. [96] to screen a large library from Specs. Drug-like filtering and cluster analysis followed the screening, and the most promising compounds of each cluster were experimentally evaluated. Two novel scaffolds were discovered; of these two, a 5-benzylidene-hydantoin derivative (see Table 5, Entry 5) was reported as a promiscuous SIRT1I (IC_50_ = 32.1 μM) and SIRT2I (IC_50_ = 37.7 μM) with promising anti-cancer potential. In addition, the newly proposed SIRT2I proved to be able to significantly cross both the artificial membrane’s GIT (gastro-intestinal tract; HDM-PAMPA) and the blood–brain barrier (PAMPA-BBB).

In 2023, Scarano et al. reported a SBVS study that exploited the abundancy of X-ray structures containing SIRT2 [66]. The authors carried out a preliminary study on the screening performances of the available X-rays while considering single- and multi-conformation procedures. In particular, the ROC-AUC associated with preliminary screening and an annotated database was reported, and the best performing conformation (PDB ID: 5Y5N) [20] was chosen to perform a VS of a CNS-focused library from ChemDIV (22,000 compounds). Five compounds were tested in vitro, where **L407-0319** (Table 5, Entry 6) was the most interesting (SIRT2 inhibition = 44%) On this basis, following computational studies on focused-VS, the discovery of pyrazole–pyrimidine derivatives with improved potency (see Compound **25** in Figure 11, as described in the following Section 4.2. Design of SIRT2Is) was enabled.

Subsequent structure-based studies on in-house compounds have led to the identification of thiazole-containing derivatives exhibiting SIRT2 inhibitory ability and selectivity toward SIRT6 [97]. The most effective one (Table 5, Entry 7) resulted in a good SIRT2I (IC_50_ = 17.3 μM).

As for SIRT1, in some cases, the structure-based approach was combined with ligand-based techniques, such as similarity search and QSAR.

In detail, in a VS study conducted in 2008, Uciechowska et al. [98] pre-filtered the Chembridge database [108] on the basis of lead-like properties and similarity with respect to **cambinol**. Then, the apo-SIRT2 crystal structure (1J8F) [47] was used for the docking of the retrieved compounds.

The candidates were selected according to their putative capability to establish a key H-bond to Gln167. In vitro tests individuated five barbiturate and thiobarbiturate derivatives with activity at the micromolar level, where the naphthyl-containing hit compound (see Table 5, Entry 8) was able to block SIRT1 (IC_50_ = 13.2 μM) and SIRT2 (IC_50_ = 9.1 μM). According to the reported docking poses, the compound displays hydrophobic interactions with PHE96 and PHE119, and H-bonds with the GLN167 and HIS187 of SIRT2.

Following the structure-based refinement of this prototype led to further analogs such as the biaryl-based thiobarbiturate being endowed with SIRT2 or SIRT1 selectivity.

The barbiturate/thiobarbiturate scaffold was exploited and optimized by the same group in 2012, including structural variations at the previously cited naphthyl group, toward other five-membered (hetero)aromatic rings [109]. This was performed by calculating the fingerprint of the most active molecules from the initial paper and using this information to screen the Chembridge database [108]. The obtained compounds were then submitted to docking in the apo-SIRT-2 active site (PDB code 1J8F) [47], as well as to other in silico and in vitro analyses. Final experimental results confirmed an improved potency of the novel derivatives (IC_50_ values in the low micromolar range). Interestingly, carrying out MD simulations on the proposed hits and collecting the corresponding predicted binding affinity values, via molecular mechanics Poisson–Boltzmann surface area (MM-PB/SA) and linear interaction energy (LIE) approaches, allowed for the development of QSAR models that explain the SIRT2 binding ability. In the case of the MM-PB/SA approach, two models, either based on ΔG or ΔH, were obtained, and the ΔH one was endowed with an excellent predictive power that gives high correlation coefficient and low RMSE values. The LIE models were also evaluated with the same dataset of SIRT2I candidates. The results revealed that the estimated binding free energies using this approach allow for obtaining better statistical parameters in terms of the predictive ability of the training set compounds than the previous MM-PB/SA model, even if those related to the test set compounds were more accurately predicted by the MM-PB/SA approach.

Moreover, the same authors applied a similar method to the discovery of new splitomicin-related SIRT2Is [99]. After a SAR investigation, the β-aryl derivatives were identified as promising splitomicin analogs. Among them, the most active compounds were submitted to fingerprint calculation, and the Chembridge database [108] was screened according to these data. A docking step in the apo-form of SIRT2 (PDB code 1J8F) [47] followed, and four candidates were selected for in vitro evaluation. The experimental study highlighted that the lactone–lactam substitution was promising, thereby leading to further optimization as the compound reported in Table 5, Entry 9 displayed an IC_50_ = 6.4 μM against SIRT2.

In 2019, Eren et al. [100] developed a LB pharmacophore from a set of thirty-one SirReal2 analogs. The pharmacophore was utilized to perform a large-scale VS on drug-like compounds from the ZINC database. Thanks to the availability of the crystal structure of SIRT2 in complex with a selective inhibitor, docking was performed in the locked-open conformation of the target (PDB code: 5DY4) [63]. Docking analysis was complemented with the MM-GBSA technique to increase the accuracy of the docking pose. Thirty-one compounds were proposed for in vitro validation and then subsequently to cluster analysis.

The two novel SIRT2 inhibitors **ZINC05417772** and **ZINC67727001** were individuated and endowed with a modest SIRT2 inhibitory ability of 84% and 73%, respectively. However, both of them were selective with respect to SIRT1,3,5 (see **ZINC05417772** in Table 5, Entry 10). Further investigations involving the bioisostere replacement of the terminal phenoxy-moiety with other heterocyclic rings allowed for an enlarging of the series of aryloxybenzamide SIRTIs, including the piridazine or pyrimidine analogs [110].

More recently, Khanfar and Alqtaishat [101] reported an integrated pharmacophore-based/QSAR model for the discovery of SIRTIs. A set of eighteen SIRT2 co-crystals was used to generate SB pharmacophore models, whose performances were evaluated through ROC curves, as described in the literature [111]. Nineteen models were selected as appropriate. In parallel, physicochemical descriptors of a set of known SIRT2Is were calculated. At this point, descriptors and pharmacophore models were integrated into a single mathematical equation by means of the Genetic Function Algorithm [112] and Multiple Linear Regression analysis [113]. The resulting integrated QSAR model evidenced three descriptors and the fit value of the most suitable pharmacophore model, which was then applied to screen the AnalytiCon Discovery database of the purified natural products [114]. Two out of the ten of the tested compounds showed an inhibition of SIRT2, with an IC_50_ in the low micromolar range (see Table 5, Entry 11). Accordingly, **asperphenamate** and **salvianolic acid B** were reported as active SIRT2Is.

Djokovic et al. tried to explore the conformational space of SIRT2 by expanding it beyond the reported X-ray structures [102]. The input conformations were retrieved by X-ray structures, the conformations derived by unbiased MD, and the conformations from MetaDynamics. For each simulated system, dynamical residue interaction network (RIN) analysis was used to reveal the functionally important residues of SIRT2 and to inspect their involvement in the conformational differences between simulated holo-complexes and the apo-system. The betweenness centrality (BC) measure was exploited as an indicator of the relevance played by each residue in the whole network. BC analysis of the binding site revealed ASP170 as one key residue in SIRT2, where it is expected to mediate the communication between different functional parts of the protein. Notably, some of the most prominent differences between the holo- and apo- forms were detected in interactions involving 135–143 and residue PHE190. This implies that the presence of ligands with different interaction patterns inside the SIRT2 binding pocket could affect the conformational behavior of the selectivity pocket.

Then, a Linear Discrimination Analysis (LDA) using FLAP 2.2.1 (Fingerprints for Ligands and Proteins) software was used to build a model with the ability to discriminate between active and inactive compounds. This model was then used to screen a library of 200,000 compounds from the SPECS database. Among the top-scored derivatives, nine candidates were selected for in vitro validation according to their belonging to an under-represented area of the chemical space with respect to the existing SIRT-2Is. **NDJ18** (Table 5, Entry 12) and **NDJ85** displayed a potency of up to 58.7 and 85.9 μM, as well as a certain selectivity with respect to SIRT1 and SIRT6 [102].

The anticancer effects of **NDJ18** were also investigated on the triple-negative breast cancer cell line, and results were obtained that indicated that this compound could represent a promising structure suitable for further evaluation.

### 3.3. Design of Dual SIRT1-2 Inhibitors via Computational Approaches

The discovery of isoform-selective SIRTIs is a challenging task as it is based on the high conservation observed in the enzyme catalytic site [25].

Consequently, sequence and structure alignment studies have indicated high similarities involving SIRTs1-3 [25], which also feature analog structural organization [26]. On this basis, efforts have been performed in the search for dual-acting SIRT1,2Is.

In 2018, Karaman et al. [115] performed a consensus SBVS of the p-ANAPL database [116] on two SIRT1 (PDB IDs 4I5I, 4ZZJ) [36,37] and four SIRT2 (PDB IDs 4R8M, 4L3O, 4RMH, 5D7P) [26,45,55,59] crystal structures to identify new promiscuous SIRT1/SIRT2Is. The hit lists from different screenings were combined, duplicates were removed, and seven compounds were tested in vitro. Two bichalcone derivatives (see the prototype in Table 6, Entry 1) were individuated as SIRT1/SIRT2Is, with an IC_50_ of around 40–50 μM). Activity toward SIRT3 was slightly inferior (20–40% inhibition).

## 4. Recently Exploited Chemical Scaffolds in the Search for SIRT1/2 Inhibitors

As a complement to the previous section, this one summarizes the main chemical scaffold recently exploited and discovered in the search of SIRT1/SIRT2Is by applying traditional medicinal chemistry such as bioisostere replacement and SAR studies.

The compounds herein discussed, as investigated from 2017, are reported and divided in SIRT1Is, SIRT2IS, dual SIRT1/2Is, and pan-SIRTIs. The main chemo-types thus far exploited are summarized in Table 7.

### 4.1. Design of SIRT1Is

**Figure 8:**
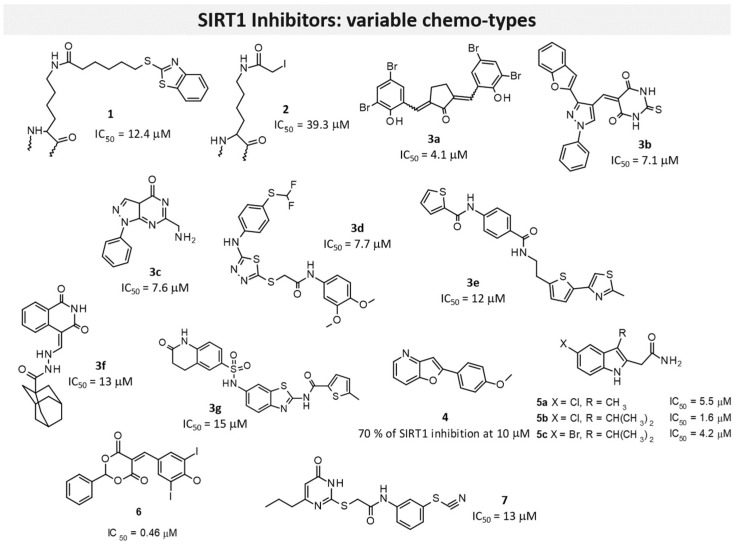
The chemical structure of SIRT1Is. Peptide-based compounds (**1**,**2**) [117], variable chemo-types screened as series **3** [81] furo-pyridine derivatives (**4**) [118], indole based (**5**) [119], benzylidene–dioxane compounds (**6**) [120], and a thiocyanate compound (**7**) [74].

In 2017, Wang and colleagues investigated bivalent SIRT1Is that were constructed by covalently linking, through a sulfur linker in some cases, the ε-amino group of the lysine of a tripeptidic scaffold to different chemical entities [117]. Some of them (i.e., Compounds **1** and **2**, Figure 8) were found to be stronger SIRT1Is (IC_50_ values of 12.4 and 39.3 μM, respectively) with a good selectivity profile. In detail, **1** bears a benzothiazole nucleus, whereas **2** is characterized by a terminal iodine atom. This study laid a foundation for the future development of bivalent inhibitors as SIRTIs [117].

In 2019, a contest-based approach was performed via collecting compound lists that were differently prioritized as putative SIRT1Is based on various computational methods [81]. This approach was managed by the authors to maximize the chance of identifying structurally diversified molecules. Following biological assays involving approximately half of the proposed compounds led to seven different SIRT1Is (see Series **3** in Figure 8).

On the basis of the pharmacological properties of furopyridine derivatives, Laxmi and colleagues recently reported different 2-substituted furo[3,2-*b*]pyridines as cytotoxic agents [118]; these were conveniently prepared with a single-pot method via sequential C-C coupling followed by C-O bond-forming reactions, and this was achieved using ultrasound irradiation in the presence of a Pd/C catalyst. All new derivatives were evaluated against MDAMB-231 and MCF-7 cell lines, and then subsequently against SIRT1. Good results were obtained: Compound **4** (Figure 8) particularly showed apoptosis-inducing potential in MCF-7 cells and a percentage of 79% SIRT1 inhibition at a 10 μM concentration [118].

In 2020, Laaroussi et al. reported the design, synthesis, and biological evaluation of a series of new indole analogs strictly related to **EX-527** [119]. In detail, they performed some structural modifications on the **EX-527** scaffold as the removal of the asymmetric carbon and the introduction of hydrophobic and bulky substituents at Position 3 of the indole was able to better interact with the hydrophobic pocket present in the active site of SIRT1. The new compounds were tested against SIRT1 and SIRT2 and evaluated for their cytotoxic activities against a panel of nine cancer cell lines. Several derivatives evidenced inhibitory activities similar to the reference compound, as well as good selectivity. Moreover, **5a**, **5b**, and **5c** (Figure 8) were found to be the most promising (IC_50_ values of 5.5, 1.6, and 4.2 μM, respectively), thereby confirming that it is possible to remove the asymmetric carbon of **EX-527** and explore the hydrophobic pocket of the active site of SIRT1 with a variety of hydrophobic substituents at the 3-position of the indole core. Interestingly, some of the derivatives that are always characterized by bulky substituents in position 3 of the indole moiety displayed an inhibition of SIRT2 and interesting cytotoxic activity, thus representing a starting point for further SAR studies on SIRT2Is [119].

In 2021, Li and colleagues identified other SIRT1Is endowed with the 5-benzylidene-2-phenyl-1,3-dioxane-4,6-dione scaffold [120]. All new synthesized compounds were evaluated for their SIRT1 inhibitory activity, with **6** (Figure 8) being found as the most potent derivative (IC_50_ of 0.46 μM). A deep investigation regarding the selectivity profile of **6** also evidenced some inhibitory activity versus SIRT5 (IC_50_ = 4.98 μM), weakly activity versus SIRT2 and SIRT3 (IC_50_ 50–120 μM), and no activity versus SIRT6. By kinetic analysis investigations, the authors also demonstrated that this inhibitor was competitive to acetyl peptide and non-competitive to NAD^+^. In addition, the interaction of the inhibitor in SIRT1 was studied by using molecular docking; finally, in vitro assays were performed to confirm the p53-increased acetylation in a concentration-dependent manner [120].

Virtual screening studies performed by Wössner on a set of thiocyanate derivatives promoted the biological evaluation of a small library of commercially available compounds [74]. Among them, a thienopyrimidone derivative (**7**) was endowed with selective SIRT1 inhibitory ability (SIRT1 IC_50_ = 13 μM; Figure 8).

**Figure 9:**
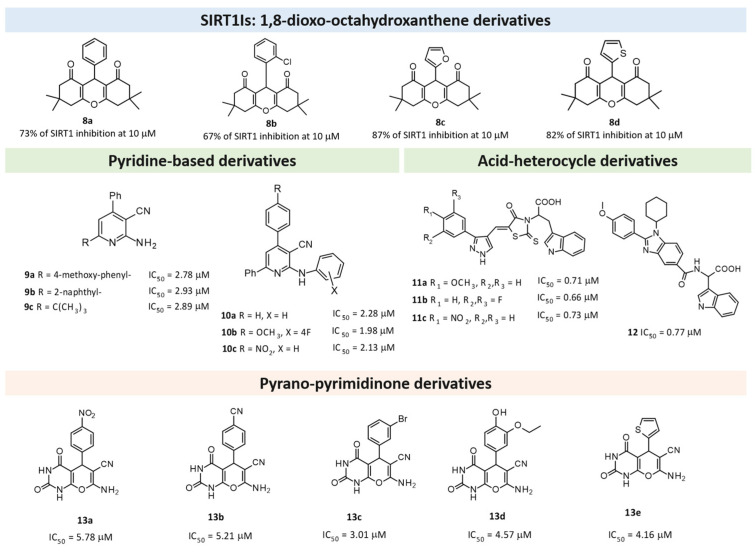
Chemical structure of the SIRT1Is, including 1,8-dioxo-octahydroxanthene derivatives (**8**) [76], pyridine-based compounds (**9**,**10**) [121,122], acid derivatives (**11**,**12**) [77], and pyrano-pyrimidinone ligands (**13**) [123].

A series of 1,8-dioxo-octahydroxanthene derivatives have been reported featuring a modest SIRT1 inhibitory ability [76]. As shown in Figure 9, the introduction of aromatic moieties tethered to the main scaffold allowed for deriving promising SIRT1Is endowed with high levels of SIRT1 percentage inhibition. The choice of the furane ring led to the most effective compound, i.e., **8c** (87% SIRT1 inhibition at 10 μM).

Challa and colleagues synthesized new pyridine derivatives, specifically 2-amino-4,6- disubstituted nicotinonitrile derivatives, as SIRT1Is [121]. These new chemical entities were obtained with good yields using an ultrasound-assisted multicomponent reaction (MCR) between suitable ketones, aldehydes, malononitriles, and ammonium acetate in the presence of Amberlyst-15 as a catalyst. In particular, the **9a**, **9b**, and **9c** compounds (Figure 9) showed interesting SIRT1 inhibition (IC_50_ ~3 μM)—better than the reference compound nicotinamide (IC_50_ ~109 μM). In silico docking studies showed a higher number of interactions than nicotinamide; in particular, amino and cyano groups formed H-bonds with ASN346 and HIS363 residues, respectively, thereby confirming the amino-nicotinonitrile scaffold as a new promising framework for the identification of SIRT1Is. Consequently, Compound **9** was identified as a potential hit for further investigations [121].

In pursuing this investigation, the same authors, using in silico framework-based drug design, designed and synthesized other pyridine compounds, which were obtained with the same synthetic method but using the sulfonic acid-functionalized Wang resin as a polymeric and recoverable acidic catalyst [122]. In detail, in this new series, in Position 2 of the pyridine nucleus, the primary amino group was substituted with a more embedded anilino substituent. SAR analysis revealed that an aryl group at the C-4 position of the central pyridine ring was the most promising over the heteroaryl or alkyl moiety, whereas an unsubstituted benzene ring was more favored at the C-6 position; similarly, an unsubstituted benzene group appeared to be better than the substituted one for the arylamino moiety at the C-2 position, with Compounds **10a**–**c** (Figure 9) being the most active. The in vitro evaluation of these three pyridine derivatives against SIRT1 revealed promising inhibitory activities (a >50% inhibition and IC_50_ values of 2.28, 1.98, and 2.13 μM, respectively). These results have been confirmed by in silico docking studies [122].

Through developments in drug design, strategically conjugating amino acid fragments with different bioactive heterocycles has proven to enhance desirable pharmacological features, such as low toxicity, high bioavailability, stability, and cell permeability [133].

In this context, Purushotham and colleagues obtained twenty-four amino acid–heterocycle conjugates using a combination of different amino acids and two types of heterocyclic scaffolds, namely benzimidazole and pyrazole [77]. In detail, the first series consisted of twelve substituted benzimidazole monopeptides derived from four amino acids (alanine, valine, leucine, and tryptophan), whereas the second one consisted of twelve substituted pyrazolyl methylidenes of the rhodanine carboxylic acids derived from four amino acids (glycine, alanine, phenylalanine, and tryptophan). All compounds were tested for in vitro enzyme-based and cell-based SIRT1 inhibition assays, and their cytotoxic activity was evaluated in both liver and breast cancer cells. In detail, tryptophan conjugates showed the best results, with the **11a**, **11b**, and **11c** pyrazole derivatives (Figure 9) and benzimidazole **12** (Figure 9) being the most active with SIRT1 inhibition in the low micromolar range (IC_50_ = 0.71, 0.66, 0.73, and 0.77 μM, respectively), which is comparable to the reference compound, **EX-527** (0.60 μM).

The authors also demonstrated that the improved SIRT1 selectivity of **11b** and **11c** over SIRT2 is probably due to the presence of strong electrostatic interactions within the two basic residues (K444 and R446) of the “SLxVxP(V/F)A” motif. This study highlights that the new **12** and **11b** compounds could represent a starting lead for designing more potent and selective SIRTIs that are useful for cancer therapy [77].

Kondabanthini and colleagues exploited the pyrano[2,3-*d*]pyrimidine framework in the search of SIRT1Is [123] by focusing on the 7-amino-5-aryl-6-cyano-5*H*-pyrano pyrimidin-2,4-dione moiety as it is endowed with anticancer properties [128] and has previously been reported as a SIRTI [134]. The authors merged pharmacophore features to achieve a SIRT1 inhibition that was previously exhibited by their pyridine-based [122] and dihydropyrano–pyrazole derivatives [135] toward Compounds **13a**–**e** (Figure 9). Among them, **13c**–**13e** were predicted as the most interesting SIRT1Is via molecular docking studies, revealing H-bonds with ILE347, ASP348, and HIS363. Additional enzymatic assays confirmed **13a**–**13e** as inhibitors, with **13c** being even more potent than nicotinamide. It also showed the effects on MCF7 and HEK 293T cell lines and the in silico-predicted favorable pharmacokinetic properties. Indeed, at the concentration of 10 μM, **13c** showed a 34 and 47% decrease in cell survival in the two previously mentioned cell lines, respectively.

### 4.2. Design of SIRT2Is

**Figure 10:**
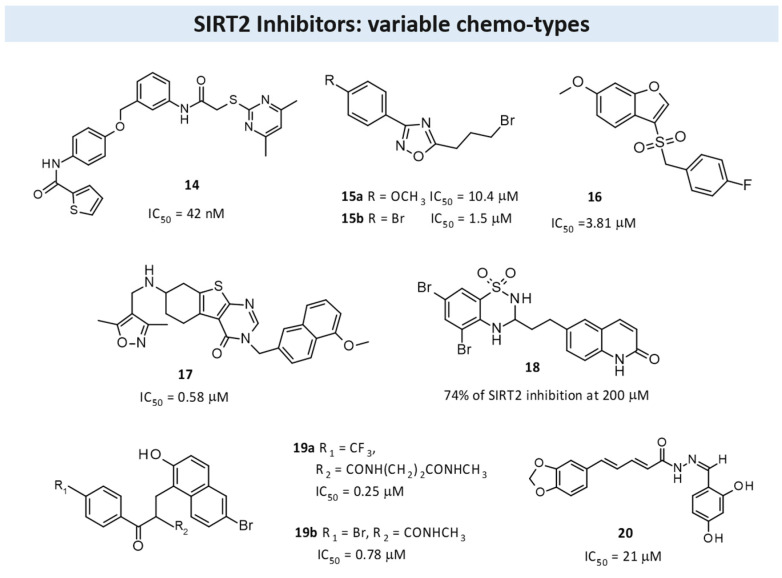
The chemical structure of SIRT2Is featuring the phenyl-acetamide chemo-type (**14**) [95], 1,2,4-oxadiazol (**15**) [65], benzofuran (**16**) [124], the thienopyrimidinone scaffold (**17**) [56], sulfonamide-containing compounds (**18**) [125], beta-keto amides (**19**) [126], and piperine–resveratrol hybrids (**20**) [127].

The pyrimidine scaffold revealed biological property activity against SIRT2 [95]. In 2011, a structure-based optimization approach led to new 2-((4,6-dimethylpyrimidin-2-yl)thio)-*N*-phenylacetamide derivatives as SIRT2Is. In detail, Compound **14** (Figure 10) showed the best pharmacological activity, with an IC_50_ value of 42 nM against SIRT2 and a very good selectivity profile. In cellular assays, **14** showed a strong antiproliferative action against the human breast cancer cell line MCF-7, and it increased the acetylation of α-tubulin in a dose-dependent manner, thus representing a new chemical entity useful for cancer treatment [95].

Other researchers have investigated the 1,2,4-oxadiazole scaffold as SIRT2Is, starting from the 1,2,4-oxadiazole hit compound previously disclosed through virtual screening procedures [65]. Extensive SAR studies have been conducted using α-tubulin-acetylLys40 peptide as the SIRT2 substrate, and they have highlighted the presence of a para-substituted phenyl ring at the C3 position of the 1,2,4-oxadiazole scaffold and the ω-haloalkyl chain at the C5 as fundamental for obtaining SIRT2 inhibition. Selected compounds (**15**; Figure 10) have showed IC_50_ values in the low μM range and are inactive up to a 100 μM concentration against SIRT1, SIRT3, and SIRT5. Their inhibition mechanism is uncompetitive toward both the peptide substrate and NAD^+^, and the crystal structure analysis in complex with SIRT2 and ADPR reveals their orientation in a still unexplored sub-cavity that is useful for further inhibitor development. In addition, **15** was found to induce apoptosis and shows good anti-proliferative activity in leukemia cell lines, and Western blot analyses confirmed the involvement of SIRT2 inhibition regarding their effects in NB4 and U937 cells. Collectively, these results provide new SIRT2Is with the 1,2,4-oxadiazole scaffold and structural insights for further inhibition improvement [65].

To obtain more potent SIT3Is, Zhou and colleagues reported a series of benzofuran derivatives [124]. Enzymatic assays have revealed that new derivatives are more able to inhibit SIRT2 with IC_50_ values at the micromolar level, with Compound **16** (Figure 10) being the most promising (IC_50_ 3.81 μM). In this way, the authors demonstrated that the benzofuran core could represent an appropriate scaffold for the development of new and drug-like SIRT2Is, and that the benzyl sulfone moiety at C3 could improve biological activity [124].

By an extensive SAR study of the thienopyrimidinone scaffold, Sundriyal identified the key pharmacophore elements needed to obtain selective SIRT2Is [56]. New synthesized compounds exert SIRT2 inhibition at sub-micromolar level, with **17** (Figure 10) being the most active and selective (IC_50_ value of 0.58 μM). The authors also reported a co-crystal structure of **17** being bound to SIRT2, thereby revealing that this class of molecules bind in an inverted fashion to what might be intuitively expected. Collectively, this information could significantly contribute to an understanding of the mechanism of action of SIRT2Is and to the identification of thienopyrimidinone analogs as an important class of selective SIRT2Is [56].

In 2020, to investigate if the bioisosteric replacement of the chroman-4-one/chromone core, which has been previously reported as potentially useful in obtaining new SIRT2Is, Seifert et al. synthesized a large library of compounds as [136] antiproliferative agents able to block SIRT2.

In detail, they investigated different less lipophilic bicyclic scaffolds to overcome the problems associated with poor physicochemical properties due to a highly lipophilic substitution pattern required for achieving a good inhibitory effect [125]. Different new derivatives based on the quinolin-4(1*H*)-one scaffold and bicyclic secondary sulfonamides or saccharins were synthesized and evaluated as SIRTIs. Among the evaluated chemo-types, the benzothiadiazine-1,1-dioxide-based compounds showed the highest SIRT2 inhibitory activity, with Compound **18** (Figure 10) being the most active (a 74% of inhibition at a 200 μM concentration). Molecular modeling studies have also been reported and have given insight into the binding mode of this new class of compounds [125].

To obtain cambinol-based SIRT2-specific inhibitors devoid of SIRT1 or SIRT3 inhibition, authors from the Fred Hutchinson Cancer Research Center (Seattle, WA, USA), who previously developed **cambinol** [15], recently investigated open-chain SIRT2Is derived from **cambinol** [126].

From a chemical point of view, new derivatives bear a functionalized beta-keto amide that makes these inhibitors susceptible to racemization under physiological conditions. For this reason, all of the compounds described herein were tested as racemates, thereby evidencing good in vitro cytotoxicity against lymphoma and epithelial cancer cell lines. In particular, **19a** and **19b** (Figure 10) showed IC_50_ values of 0.25 μM and 0.78 μM against SIRT2, with good selectivity against SIRT1 and SIRT3; in addition, in B-cell lymphoma cells, they evidenced the apoptotic effect and strong anti-proliferative properties [126].

In 2021, a series of novel piperine–resveratrol hybrids endowed with interesting anti-proliferative action against leukemia HL-60 (TB) and breast cancer MDA-MB-468 have been evaluated by Tantawy [127]. The results of a screening regarding SIRT inhibition evidenced a specific inhibitory activity and selectivity against SIRT2 than SIRT1 at a 5 μM and 50 μM concentration, with Compound **20** (Figure 10) being the most promising (IC_50_ = 21 μM) and comparable to the reference compound, **AGK2**. Additional molecular docking investigation indicated that **20** fits nicely inside both the extended C-pocket and selectivity pocket, and it could compete with the substrate acyl-Lys, whereas another possible binding pattern showed that **20** could act by the partial occlusion of the NAD^+^ C-pocket. Collectively, this study significantly improves the knowledge regarding the SIRT2 inhibition mechanism, and it could be useful for the development of a new generation of SIRT2Is [127].

**Figure 11:**
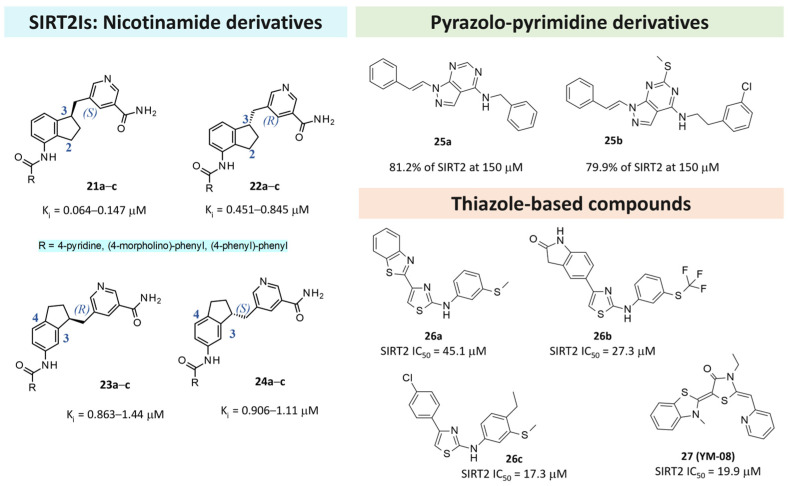
The chemical structure of SIRT2Is exhibiting the nicotinamide core (**21**–**24**) [128], the pyrazolo–pyrimidine scaffold (**25**) [66], and the thiazole main ring (**26**, **27**) [97].

In 2023, Ai and colleagues described novel 3-aminobenzyloxy nicotinamide derivatives endowed with an SIRT2 inhibitory ability via the development of constrained analogs [128]. The authors referred to their previous published data about 5-aminonaphthalen-1-yloxy nicotinamide [137] and 3-aminobenzyloxy nicotinamide derivatives [138] as SIRT2Is to guide further SAR analysis, including the development of the constrained **21**–**24** analogs (Figure 11) [128]. Based on the following biological assays, the 2,3-constrained isomers (**21**,**22**) were preferred to the 3,4-constrained ones (**23**,**24**). Furthermore, the 2,3-constrained (*S*)-isomers proved to be the most promising.

Scarano and colleagues described deepened virtual screening studies performed on thirty-seven PDB codes involving SIRT2 alone or in the presence of different chemotypes [66]. A set of six enzyme–inhibitor complexes (5Y5N, 4RMH, 5D7Q, 5MAR, 5MAT, and 5YQO) were assessed in their ability to filter active/inactive compounds from a benchmarking database that includes 2% of active compounds. The selected computational protocol was applied for the search of putative SIRT2Is based on the ChemDIV library (22, 000 compounds), thereby leading to **L407-0319** as a modest inhibitor (see previous Table 5, Entry 6). Based on the SIRT2 inhibitor requirement exploited in the literature, the introduction of a central bicyclic hetero-aromatic ring and the presence of two aromatic terminal groups allowed for the SIRT2 inhibitor ability to be exhibited. Accordingly, a set of in-house pyrazolo–pyrimidine derivatives (**25**) were evaluated in silico and then in enzymatic assays to ascertain their ability to function as SIRT2Is. As shown in Figure 11, **25a**,**b** proved to be interesting for SIRT2 inhibition [66].

Recently, Abbotto reported structure-based studies performed based on the X-ray data of 4RMG and 5MAT, where the focus was on the (flexible) SirReal2 chemo-type and (rigid) thieno-pyrimidinone scaffold. The results allowed for guiding the in silico screening of further thiazoles (**26,27**) that exhibited SIRT2 inhibitor behavior [97]. Indeed, preliminary in silico screening at both 4RMG and 5MAT, while maintaining the protein binding site as rigid or flexible, led to comparable docking poses with those of the co-crystallized ligand experimental data. Among the studied compounds, **26a**-**c** and **27** (Figure 11) were the most effective (SIRT2 IC_50_ = 17.3–45.1 μM). Interestingly, **27** (**YM08**) was also reported as a heat-shock protein 70 (HSP70) inhibitor [139].

**Figure 12:**
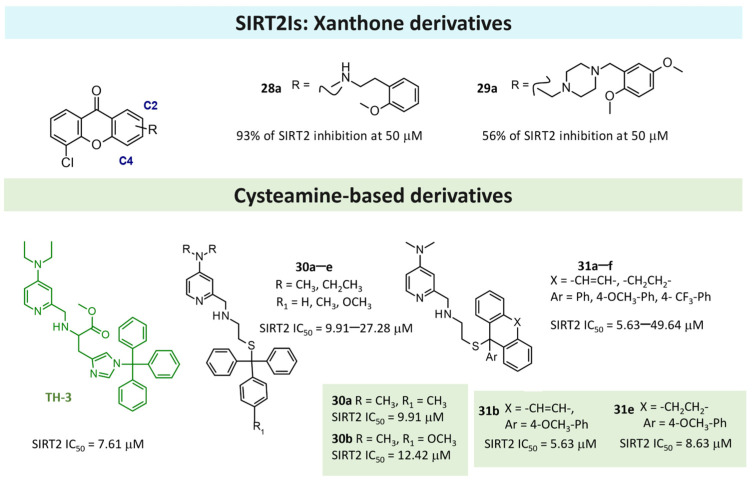
The chemical structure of SIRT2Is, including xanthone derivatives (**28**,**29**) [80] and cysteamine-based compounds (**30**,**31**) [130]. The reference compound (**TH-3**) in the development of cysteamine derivatives is highlighted in green.

Very recently, some of the natural xanthone-based analogs present in *Garcinia mangostana* were proven as acting as SIRTIs [80]. In particular, **γ-mangostin** was shown to inhibit SIRT2 (SIRT2 IC_50_ = 3.8 μM) and also SIRT1, SIRT3 (SIRT1 IC_50_ = 22.4 μM), and SIRT3 IC_50_ = 26.8 μM) [140]. On this basis, Mazur and colleagues reported six xanthone derivatives as SIRT2Is (**28**,**29**), which exhibited a secondary (**28**) or a tertiary (**29**) amine group tethered to the C2 or C4 carbon atoms of the main scaffold, respectively [129]. Among them, three secondary amine analogs (**28a**–**28c**) showed an SIRT2 inhibitory ability of 82–93% (at 50 μM), while three tertiary amine compounds (the **29a**–**29c** piperazines) showed an SIRT2 inhibition of 48–56%, with **28a** and **29a** being the most promising of the two series (see Figure 12).

In 2024, Bradan and colleagues described the rational design of cysteamine derivatives [130] based on previous analogs featuring a main diethyl amino-pyridine ring tethered to a central histidine core (the **TH-3** compound; Figure 12) [141]. Initially, the authors reported the aforementioned pyridine and histidine groups as capable of occupying the selectivity pocket and the substrate one as describable by the reference SIRT2I, SirReal2. This information suggests further SAR expansion through the design of cysteamine-based analogs that exhibit a flexible group in place of the previous histidine ring (**30**, **31**) or via locking two phenyl rings, as featured by **31**. Among the two series of derivatives, the choice of the dimethyl aminopyridine substituent led to more potent compounds than those bearing the diethethylaminopyridine ring (see **30a** and **30b** in Figure 12). The locked conformation compounds (**31**) showed interesting results, with the 4-OCH_3_-phenyl substituent compounds being the best SIRT2Is (see **31b** and **31e** in Figure 12). In addition, restrictions with un-saturated or saturated linkers via tricyclic rings, as shown by **31** turns, resulted in an improvement in the SIRT2 IC_50_ values. Accordingly, the optimized **31b** and **31e** compounds were more promising than the previously mentioned **30a** and **30b** compounds.

### 4.3. Design of Dual SIRT1/2Is

**Figure 13:**
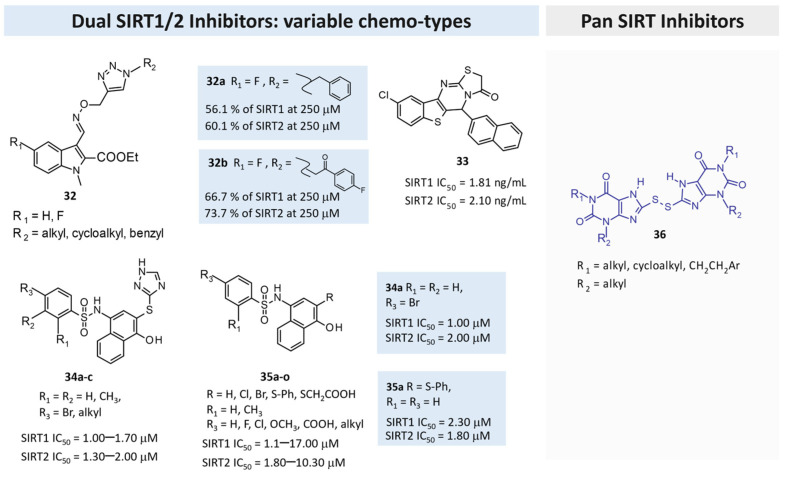
The chemical structure of dual SIRT1/2Is featuring the oxime motif (**32**) [104], the benzothieno[3,2-*d*]pyrimidine core (**33**) [131], and the naphthyl-based compounds **34**,**35** [78]. The pan SIRTIs bearing the 8-mercapto-3,7-dihydro-1*H*-purine-2,6-dione core (**36**) are highlighted in violet [132].

In 2019, Manjula et al. attempted to integrate, through an oxime ether link, the 1,2,3-triazole unit (which is endowed with different pharmacological properties) to the position-3 of the indole ring, which is known as a scaffold that is useful for obtaining SIRTIs [104]. It was envisaged that the introduction of an oxime functionality might enhance the potency of a molecule due to their extensive coordinating capability (via its binding affinity with the receptor site) and pharmacological activity. In this way, the authors synthesized twenty-two hybrid molecules by a click chemistry approach (the **32** derivatives, Figure 13). In vitro binding and deacetylation assays were carried out to characterize their inhibitory effects against SIRT1 and SIRT2. Four of the derivatives resulted in specific SIRT1Is, with three specific SIRT2Is and two dual SIRT1/2Is (**32a** and **32b**). Collectively, these compounds open up newer avenues for exploring the specific inhibitors of SIRT1 and SIRT2, with therapeutic implications for humanpathologies, including many age-related diseases [104].

In 2020, a series of benzothieno[3,2-*d*]pyrimidines, which are active as anticancer agents from a large screening performed by National Cancer Institute (NCI, USA), was investigated by Khalil [131]. Novel derivatives have been designed by modifying the tetrahydrobenzo[4,5]thieno[2,3-*d*]pyrimidine scaffold, which has previously resulted in active antiproliferative agents. This was conducted to study the effect of chemical modifications and to explore thieno[2,3-*d*]pyrimidines as a potent SIRTI. Most of the tested compounds showed good inhibitory activity against the MCF-7 breast cancer cell line and the UO-31 renal cancer cell line in the range of 17.88–68.65% from a single dose (10^−5^ M).

The investigation of the isoform selectivity proved that Compound **33** (Figure 13) showed higher selectivity against the SIRT1 and SIRT2 enzymes (IC_50_ = 1.81 and 2.10 mg/mL, respectively, with 6.6 being more potent than **cambinol**, which was used as the reference compound) than SIRT3 enzyme (IC_50_ = 20.5 mg/mL). In addition, **33** evidenced a stronger activity than **cambinol** in the hyperacetylation of the α-tubulin protein. A molecular docking study into the SIRT2 active site was performed; the naphthyl group of **33** is particularly supposed to provide better accommodation in the hydrophobic cleft by forming favorable hydrophobic interactions with the binding site in the same manner as previously reported by SIRT2Is [142]. Collectively, biological results have proven that the **33** tetracyclic derivative is a privileged scaffold for the design and discovery of novel anticancer agents that are able to block SIRT1 and SIRT2 [131].

Based on SBVS strategies constructed via the SPECS database, Cai and colleagues recently discovered the hit compound **hsa62** as a naphthyl-based SIRT1/2 inhibitor (see previous Table 4, Entry 10) [78]. This information has paved the way for the following search of the substructure of further naphthyl-based congeners in the SPECS compound database. Thus, twenty-six analogs were selected and purchased for the second round of bioactivity evaluation. Among them, Compounds **34** and **35** have been evaluated in enzymatic assays of SIRT1,2,3. As shown in Figure 13, some derivatives have proven to be promising dual SIRT1/2 inhibitors, thereby confirming the choice of the triazolyl-based or S-phenyl ring at the naphthyl position 3 as one of the most effective. On the other hand, the introduction of halogen atoms at the phenyl R_3_ group leaves the other R_1_ and R_2_ as unsubstituted, which is a preferrable result. Accordingly, **34a** and **35a** were found to be the most interesting derivatives within these series.

### 4.4. Design of Pan SIRTIs

Interestingly, other authors in 2020 identified new pan-SIRTIs based on the scaffold of 8-mercapto-3,7-dihydro-1*H*-purine-2,6-dione (Compound **36**, Figure 13), which are able to block SIRT1, SIRT2, SIRT3, and SIRT5 with different potency in the low micromolar range [132]. In detail, the compounds are dimers linked by a disulfuric bond.

By molecular modeling studies focused on the binding modes of the inhibitors with SIRT3, the authors hypothesized that the new synthesized derivatives occupy the acetyl lysine binding site and interact with SIRT3 mainly through hydrophobic interactions. The binding mode was validated by a site-directed mutagenesis of SIRT3 and SAR analysis. Consistently, the subsequent enzyme kinetic assays and microscale thermophoresis investigations showed that these compounds are competitive inhibitors to the acetyl substrate and mix-type inhibitors to NAD^+^. Collectively, these results provide new promising hits for the development of more potent pan-SIRTIs [132].

## 5. Conclusions

This review summarizes the successful computational studies thus far described in the literature that have led to the discovery of new hit compounds in the search for SIRT modulators. During the last few years, several types of experimental data about different SIRT isoforms have become available, including apo-conformation or enzyme–ligand complexes. The presence of substrates and/or co-factors have allowed for a better exploration of the ligand-binding event, thereby facilitating the search for novel potent ligands. A perspective of the available structural information about SIRT1 has indicated three and five types of SIRT1 X-ray crystallographic data in the apo- and in the holo-conformations. The data on the binding process to SIRT1 activators is better represented than that of enzyme inhibitors, with **EX-527** being the most exploited compound.

Regarding SIRT2, fourteen sources of X-ray crystallographic data have reported enzyme apo-conformation, while twenty-three SIRT2/inhibitor complexes have also been described. Among them, the presence of the well-known selective SIRT2I SirReal2 and its highly related analogs has been investigated. Based on the evidence, most of the applied computational strategies have relied on SBVS methods toward variable chemo-types alone or via combined SBVS-LBVS ones. In the last section of this review, a perspective (from 2017) of the most exploited chemical scaffolds in the design and optimization of SIRT1/2Is are reported, and the higher structural variations that have been found in the search of SIRT2 ligands than that of SIRT1 ligands are highlighted. This review has highlighted the possibility of efficiently setting up pharmacophore models as an additional tool in the rational design of future new modulators.

## 6. Future Perspectives

SIRTs are NAD^+^-dependent enzymes playing an important part in the pathogenesis and treatment of various disorders [3]. They are involved in several cellular activities like DNA repair, cellular metabolism, mitochondrial function, inflammation [143], and oxidative stress, which characterize many chronic diseases, like diabetes, cancer, cardiovascular, osteoporosis, and neurodegenerative diseases.

Over the years, various SIRT family members have been identified (SIRT1-7), and their roles have been thoroughly investigated [144]. Among them, SIRT1 and -2 represent the most-studied SIRTs under a pharmacological perspective. In cancer, SIRT1 has a controversial role, with oncopromoter and oncosuppressor functions being reported [9], while treatment with SIRT1 modulators for neurodegenerative diseases has proven to be advantageous [145,146]. Regarding SIRT2, inhibitors have been reported as anti-proliferative agents [87], or they have been investigated as putative neuroprotective agents in neurodegenerative disorders [147].

Based on the SIRT involvement present in many of the biological processes investigated above, many research groups and laboratories have attempted to develop both SIRT1/2 activators and inhibitors. Both of the two series of compounds represent useful pharmacological tools to better investigate the role of SIRTs in different physiological or pathological events, as well as in guiding the development of effective therapeutic agents.

Several efforts to develop SIRT activators or inhibitors have been managed by relying on natural products and related analogs, as well as via small molecule inhibitors or mechanism-based peptide modulators [148,149,150,151]. However, the structural similarity within SIRTs often complicates the rational design and development of selective ligands, especially for SIRT1-3. In particular, the SIRT inhibitors binding at the highly conserved interface between the zinc-binding domain and the Rossmann fold makes the development of selective SIRTIs a challenging task. Nevertheless, for both SIRT1 and SIRT2, a few selective inhibitors have been reported. As a further advantage, the partially selective SIRT1 inhibitor **EX-527** has been co-crystallized within the enzyme active site, thereby providing important information for selectivity rationalization. The case of SIRT2 is even better explored, with fourteen selective small molecules belonging to six distinct chemo-types that have been co-crystallized with the target. The large quantity of structural data provides an unprecedented possibility for exploring SIRT1/2 selective drug design. However, the protein rearrangements and induced-fit effect responsible for SirReal2 selectivity, which have been investigated via structure-based studies, are not easily predictable a priori for novel chemo-types. Indeed, additional structural studies, as well as the use of computational techniques involving protein dynamics (such as MD), may help in a further exploration of SIRT1/2 dynamics, and they may also pave the way for more selective inhibitors. A deepened comparison of the protein-binding sites can be performed, including different co-crystallized ligands to achieve more information on the following: (i) the most flexible SIRT domains and (ii) the main different residues involved in the ligand binding. On the other hand, it should be noticed that structural information is still missing for SIRT4, which cannot be included in the active site comparison. Therefore, the development of specific SIRT activators and inhibitors exhibiting high selectivity values within SIRT1-2 isoforms still represents an urgent need for clarification with respect to the specific physiological pathways involving SIRTs, as well as the consequent relevant role in several pathologies. This could be managed by applying ligand-based methods such as QSAR analysis and pharmacophore modeling in the case of a high number of collected ligands. In addition to selectivity, the potency of SIRTIs could be improved. It has to be noticed that just a few SIRT1/2 inhibitors exhibit IC_50_ values in the nanomolar range, thus making the structural optimization of variable chemo-types still manageable. Among the most potent SIRT1/2 inhibitors, we can find the thieno[3,2-d]pyrimidine-6-carboxamides class, which reaches potency values in the low nanomolar range for SIRT1-3 [38]. Additionally, substrate-based inhibitors can be better investigated. Despite the classical limitation of peptide-based drugs, recent advances in drug delivery and peptide stabilization represent an opportunity to keep on exploring this area [152].

As previously mentioned, computational studies are expected to allow the design and hit-to-lead optimization process of numerous chemo-types, and they also promise to facilitate the drug design process, even in the case of the most innovative approaches. In this context, Schiedel et al. experimented with the first SIRT2 PROTAC [153,154]**,** thereby unveiling a novel promising pathway for SIRT-targeting drugs. Apart from the relevance in drug design, which has been extensively proven by a plethora of studies leading to novel SIRT1/2 modulators, the use of computational techniques can complement experimental data in the attempt to clarify the molecular mechanism and dynamics of this class of enzymes. A key example of this is the in silico investigation of the Sirtuin Activating Compounds (STAC)-based mechanism that is used to evoke SIRT1 activation, which was explored in several computational studies [40,41,42,43,44]. Other (non-exhaustive) examples of computational techniques applied to the study of SIRTs help determine the use of MD simulations to investigate conformational rearrangements upon ligand and substrate binding [155], clarify the intramolecular inhibition mechanism [156], investigate the molecular mechanism of SIRT catalysis via QM/MM simulations [157], or investigate its energetic profile [158]. Essential dynamic techniques were utilized to explore the structural determinants of SIRT1-3 activity and selectivity [159], among others.

Along with this, the machine-learning-based tool, namely SIRT2i_Predictor, has been developed, thus providing further support for the conventional VS calculations and for the lead optimization process [160]. This was proposed based on a panel of machine-learning regression and classification-based models to predict ligand potency and selectivity toward SIRT1-3. The possibility of an inspection of molecule fragments bearing pharmacophore features for SIRT2 binding is thought to be an additional tool that supports lead-optimization campaigns as complementary to the traditional SIRT1/2 structure-based approaches. To conclude, better activity and selectivity profiles would be required to clarify the intricate pharmacology of SIRT1/2 and to achieve drug-like features. Molecular modeling techniques have significantly contributed to the discovery of novel compound targeting SIRT1/2 and to hit-to-lead optimization, as diffusely presented in the previous paragraph. In addition, the computational approach was employed to clarify the mechanistic aspect of the general target behavior, thus representing an important tool for the general knowledge of this important molecular target.

## Figures and Tables

**Table 1:** The X-ray crystallographic structures of *h*SIRT1 obtained via X-ray diffraction. Protein structures in the apo- or holo-form are reported in cyan and gray, respectively. The resolution (R; in Å), release date (R.D.), the corresponding reference (Ref.), and the protein length as number of residues (L) are reported.

PDB ID	Structure Title	Small Molecule-like Ligand (Selectivity)	Cofactor	Substrate	R (Å)	R.D.	Ref.	L	Additional Domains/ Regions
4IG9	Structure of NAD^+^-dependent protein deacetylase sirtuin-1 (open state)	None	None	None	2.64	2013	[27]	281	C-terminal regulatory segment (CTR)
4KXQ	Structure of NAD^+^-dependent protein deacetylase sirtuin-1 (closed state)	None	ADPR	None	1.849	2013	[27]	281	C-terminal regulatory segment
4IF6	Structure of NAD^+^-dependent protein deacetylase sirtuin-1 (closed state)	None	ADPR	None	2.25	2013	[35]	281	C-terminal regulatory segment (CTR)
5BTR	Crystal structure of SIRT1 in complex with resveratrol and an AMC-containing peptide	**Resveratrol** (activator) 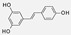	None	p53-AMC peptide	3.2	2015	[34]	397	N-terminal domain
4I5I	Crystal structure of the SIRT1 catalytic domain bound to NAD^+^ and an EX527 analog	**EX-527** analog (inhibitor)(PS) ^†^, 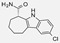	NAD^+^	None	2.5	2013	[36]	287	
4ZZI	SIRT1/Activator/Inhibitor Complex	(**4TQ**) activator 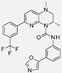 (**1NS**) inhibitor (NS ^††^) 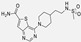	None	None	2.73	2015	[37]	356	N-terminal domain, CTR
4ZZH	SIRT1/Activator Complex	Activator 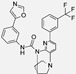	None	None	3.10	2015	[37]	356	N-terminal domain + C-terminal regulatory segment (CTR)
4ZZJ	SIRT1/Activator/Substrate Complex	Activator 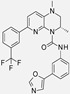	Carba-NAD^+^	Ac-p53	2.74	2015	[37]	356	N-terminal domain

^†^ PS = partial selectivity (nM on SIRT1 and μM on SIRT2) and ^††^ NS = non-selective [38]. Adenosine diphosphate ribose (ADPR), nicotinamide adenine dinucleotide (NAD^+^), and carba-nicotinamide adenine dinucleotide (Carba-NAD^+^) are listed, if present.

**Table 2:** The crystal structures of *h*SIRT2 in apo-conformation as derived by X-ray crystallography. The resolution (R; in Å), release date (R.D.), the corresponding reference (Ref.), and the protein length as the number of residues (L) are reported. The PDB ID code is highlighted in cyan.

PDB ID	Cofactor	Substrate (Intermediate/Reaction Product)	R (Å)	R.D.	Ref.	L	PDB ID	Cofactor	Substrate (Intermediate/Reaction Product)	R (Å)	R.D.	Ref.	L
1J8F	None	None	1.70	2001	[47]	323	4X3O	None	1′-SH-2′-O-myristoyl ADPR (covalent intermediate)	1.50	2016	[48]	304
3ZGO	None	None	1.63	2013	[28]	325	5FYQ	None	13-mer trifluoroacetylated Ran peptide (modified substrate)	3.00	2016	[46]	360
3ZGV	ADPR	None	2.27	2013	[28]	325	5G4C	Carba-NAD^+^	4-oxononanoyl peptide	2.10	2017	[49]	323
5D7O	ADPR	None	1.63	2015	[26]	310	6L66	NAD^+^	H3K18 myristoylated peptide	2.17	2020	[50]	304
4Y6Q	None	2′-O-myristoyl-ADP-ribose (reaction product), None	1.90	2016	[51]	293	6L65	None	H3K18 myristoylated peptide	1.80	2020	[52]	307
4Y6L	None	H3K9myr peptide	1.60	2016	[51]	293	6L71	None	Reaction intermediate I and II (c.ADPR and NAM)	2.11	2021	[53]	304
4Y6O	None	Myristoylated peptide (TNF-alphaK20myr)	1.60	2016	[51]	293	6L72	None	Final product (NAD^+^-condensed Ac-R)	2.50	2021	[54]	304

**Table 3:** The crystal structures of *h*SIRT2 in the presence of protein inhibitors, as derived by X-ray crystallography. The resolution (R; in Å), release date (R.D.), the corresponding reference (Ref.), and the protein length as the number of residues (L) are reported. The PDB ID code is highlighted in cyan. The selective (S) and unselective (NS) ligands are also noted.

PDB ID *(R)*	Inhibitor (Selectivity)	Cofactor/ *Substrate*	R.D.	Ref.	L	PDB ID	Inhibitor (Selectivity)	Cofactor/ *Substrate*	R.D.	Ref.	L
4L3O *(R = 2.52)*	Macrocyclic peptide S2iL5 (inhibitor) (NS)	None	2013	[55]	302	5MAT *(R = 2.07)*	Thienopyrimidinone-inhibitor (S) 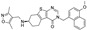	None	2017	[56]	303
4RMG *(R = 1.88)*	(**SirReal2**) (S) 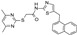	NAD^+^	2015	[45]	304	5Y5N *(R = 2.30)*	Anilinobenzamide derivative(S) 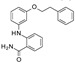	None	2017	[20]	336
4RMJ *(R = 1.87)*	Nicotinamide 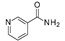	ADPR	2015	[45]	304	5YQM *(R = 1.74)*	**A29**-inhibitor (S) 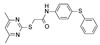	None	2018	[57]	306
5D7P *(R = 1.76)*	**EX-243** (NS) 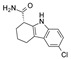	ADPR	2015	[26]	304	5YQO *(R = 1.48)*	**L5C**-inhibitor (S) 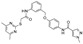	None	2018	[57]	306
4RMI *(R = 1.45)*	(**SirReal1**) (S) 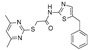	None *Ac-Lys-OTC peptide*	2015	[45]	304	5YQL *(R = 1.60)*	**A2I**-inhibitor (S) 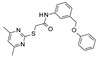	None	2018	[57]	306
4RMH *(R = 1.42)*	(**SirReal2**) (S) 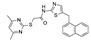	None *Ac-Lys-H3 peptide*	2015	[45]	304	5YQN *(R = 1.60)*	**L55**-inhibitor (S) 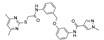	None	2018	[57]	306
5D7Q *(R = 2.01)*	inhibitor, (**CHIC35**) (PS) 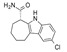	ADPR	2015	[26]	304	5Y0Z *(R = 2.00)*	**NPD11033**-inhibitor (S) 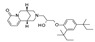	None	2018	[58]	293
4R8M *(R = 2.10)*	BHJH-TM1 (thiomyristoyl peptide-mechanism-based inhibitor)	None	2015	[59]	319	6QCN *(R = 2.23)*	**Quercetin**	ADPR	2019	[60]	304
5DY5 *(R = 1.95)*	Sirt2-IN-1 (S) 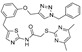	None	2016	[61]	304	6NR0 *(R = 2.45)*	Glucose-TM-1beta glucose-conjugated Thiomyristoyl lysine (mechanism-based inhibitor), covalently bound to cofactor	ADPR (fused to glucose TM-1beta)	2020	[62]	319
5DY4 *(R = 1.77)*	SirReal2 analog (S) 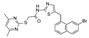	NAD^+^	2016	[63]	304	7BOS *(R = 1.7)*	Myristoyl thiourea inhibitor n. 13 (mechanism-based inhibitor)	None	2021	[64]	293
4X3P *(R = 1.80)*	Peptide-like inhibitor (BHJH-TM1)	Carba-NAD^+^	2016	[48]	304	7BOT *(R = 1.7)*	Myristoyl thiourea inhibitor, No.23 (mechanism-based inhibitor)	None	2021	[64]	293
5MAR *(R = 1.89)*	(Oxadiazole inhibitor) (S) 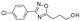	ADPR	2017	[65]	303						

**Table 5:** A perspective of the successful computational studies applied in the search of SIRT2 modulators. Activators (A) and inhibitors (I) are listed. Structure-based virtual screening (SBVS) studies and ligand-based (LB) ones or LB/SBVS are highlighted in light cyan and light yellow. The release date (R.D.) and the corresponding reference (Ref.) are reported.

Entry	R.D.	Ref.	Type of Computational Method	Selectivity over Other Isoforms	Screened Database (n. of Compounds)	Most Active Compound/Proposed Compound	Activator/Inhibitor	Potency
1	2004	[91]	Queries: (SB) features calculated on MD generated conformation + Docking	Not tested	Maybridge library	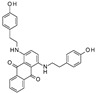	I	56.7 μM (IC_50_)
2	2006	[93]	Queries: (SB) features calculated on MD generated conformation + Docking	Not tested	Maybridge Screening Collection and LeadQuest libraries	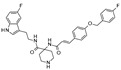 Indole derivative	I	51 μM (IC_50_)
3	2010	[94]	SBVS	1	NCI Diversity Set II	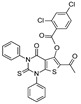 Nucleoside analog (thieno[2,3-d]pyrimidine)	I	8.7 μM (IC_50_)
4	2011	[95]	Multi-target SBVS	3,5,6	NCI diversity set (1990)	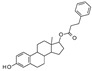 **CSC8**	I	4.8 μM (SIRT2 IC_50_)
5	2016	[96]	SBVS	1	SPECS library (197,477)	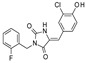 5-benzylidene-hydantoin	I	37.7 μM (IC_50_)
6	2023	[66]	SBVS	Not tested	ChemDIV (22,000)	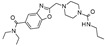 **L407-0319**	I	44.3% inhibition at 150 μM
7	2023	[97]	SBVS	6	In-house library	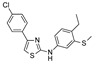	I	17.3 μM (IC_50_)
8	2008	[98]	LB: similarity based	1	Chembridge database (~328, 000)	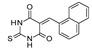 Thiobarbiturates	I	9.1 μM (IC_50_)
9	2008	[99]	LB: similarity based	Not tested	Chembridge (~328,000)	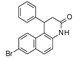 Lactame analogs of beta-phenylsplitomicins	I	6.4 μM (IC_50_)
10	2019	[100]	LB: pharmacophore based	1,3,5	ZINC drug-like database (13,000,000)	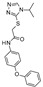 **ZINC05417772**	I	84.28% inhibition at 300 μM
11	2021	[101]	SBVS pharmacophore model combined with (SB+LB) QSAR	Not tested	AnalytiCon Discovery database of purified natural products (5637)	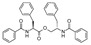 **Asperphenamate**	I	1.94 μM (IC_50_)
12	2022	[102]	SBVS on a linear model combining X-rays, MD-derived conformations, and MetaD-derived conformations	1,6	SPECS (200,000)	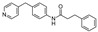 **NDJ18**	I	58.7 μM (IC_50_)

**Table 6:** A perspective of successful computational studies applied in the search of dual-SIRT modulators. Activators (A) and inhibitors (I) are listed. The release date (R.D.) and corresponding reference (Ref.) are reported.

Entry	R.D.	Ref.	SIRT(s)	Type of Computational Method	Selectivity over Other Isoforms	Screened Database (n. of Compounds)	Most Active Compound/Proposed Compound	Activator/Inhibitor	Potency
1	2018	[115]	1,2	SBVS on different conformations of sirt-1 and sirt-2	3	Pan-African Natural Products Library (463)	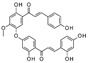	I	40.8 μM (SIRT1 IC_50_) 44.8 μM (SIRT2 IC_50_,)

**Table 7:** The chemical scaffolds reported as SIRT1, SIRT2, dual SIRT1/2, and pan-SIRT inhibitors from 2017.

Type of Inhibitors	Chemical Scaffold	References
SIRT1Is	Tripeptide linked to heterocyclic nuclei	[117]
	Furopyridine derivatives	[118]
	Indole compounds (strictly related to **EX-527**)	[119]
	Benzylidene–dioxane compounds	[120]
	Pyridine derivatives	[121,122]
	Tryptophan conjugates	[77]
	Pyrano[2,3- d ]pyrimidinone derivatives	[123]
	Variable five-membered ring derivatives	[81]
	Thienopyrimidone derivatives	[74]
	1,8-dioxo-octahydroxanthene derivatives	[76]
SIRT2Is	Pyrimidine derivatives	[95]
	1,2,4-oxadiazole substituted analogs	[65]
	Benzofuran derivatives	[124]
	Thienopyrimidinone compounds	[56]
	Benzothiadiazine-1,1-dioxide-based compounds	[125]
	Cambinol-related compounds	[126]
	Piperine–resveratrol compounds	[127]
	Nicotinamide-containing compounds	[128]
	Pyrazolo–pyrimidine derivatives	[66]
	Thiazole-based compounds	[97]
	Xanthone derivatives	[129]
	Cysteamine derivatives	[130]
Dual SIRT1/2Is	Indole and triazole derivatives,	[104]
	Benzothieno[3,2-d]pyrimidines,	[131]
	Naphthyl-based compounds	[78]
Pan-SIRTIs	8-mercapto-3,7-dihydro-1*H*-purine-2,6-dione derivatives	[132]

## Data Availability

Data sharing not applicable.
